# Mesenchymal stroma drives axonogenesis and nerve-induced aggressiveness in osteosarcoma

**DOI:** 10.1186/s13046-025-03532-2

**Published:** 2025-09-30

**Authors:** Gemma Di Pompo, Thimios A. Mitsiadis, Pierfrancesco Pagella, Alessandro Pasquarelli, Giuliano Bettini, Silvia Sabattini, Alberto Righi, Sofia Avnet, Nicola Baldini

**Affiliations:** 1 Biomedical Science and Technologies and Nanobiotechnology Laboratory, IRCCS Istituto Ortopedico Rizzoli, 40136 Bologna, Italy; 2 Orofacial Development and Regeneration, Institute of Oral Biology, Center for Dental Medicine, University of Zürich, Zürich, 8032 Switzerland; 3 Foundation for Research and Technology-Hellas (FORTH), University of Crete, Heraklion, 70013 Greece; 4 Department of Biomedical and Neuromotor Sciences, University of Bologna, 40138 Bologna, Italy; 5 Department of Veterinary Medical Sciences, University of Bologna, 40064 Ozzano dell’Emilia, Italy; 6 Department of Pathology, IRCCS Istituto Ortopedico Rizzoli, 40136 Bologna, Italy; 7 Present Address: m2Lab, Division of Biophysics and Bioengineering, Department of Physics, Chemistry and Biology (IFM), Linköping University, Linköping, Sweden

**Keywords:** Osteosarcoma, Nerves, Mesenchymal stromal cells, Interleukin-6, Brain-derived neurotrophic factor, Axonogenesis, Tumour microenvironment

## Abstract

**Background:**

Osteosarcoma (OS), the most common primary bone malignancy, is a leading cause of cancer-related mortality in children and adolescents. Besides genomic abnormalities, several features of tumour microenvironment (TME), including cancer-associated mesenchymal stromal cells (MSC), have been recognized to play a key role in OS progression. The pathogenetic function of *de novo* innervation in TME has been extensively studied in carcinomas but is still an unexplored area of investigation in sarcomas, including OS.

**Methods:**

We evaluated nerve infiltration in tissue samples from a small cohort of human OS (*n* = 5) and from canine OS (*n* = 11), a translational model for the human disease, by βIII-tubulin immunostaining. We then analysed nerve-stroma-tumour crosstalk using direct and indirect co-cultures of dorsal root ganglion (DRG) neurons with OS/tumour-associated mesenchymal stromal cells (MSC and cancer-associated fibroblasts, CAF), both under standard and microfluidic conditions. In particular, we investigated the effects of tumour and stromal cells on axonal tropism and outgrowth by measuring neurite recruitment, length, and branches and, vice versa, the impact of neuron-derived secretome on OS cell proliferation and migration. Finally, we assessed the secretion of pro-neurotrophic mediators, including brain-derived neurotrophic factor (BDNF), interleukin-6 (IL-6), and nerve growth factor (NGF), by MSC, CAF, and OS cells. The functional roles of IL-6 and BDNF were also verified by the blocking antibody Tocilizumab (TCZ) and the neutralizing Anti-BDNF antibody.

**Results:**

We provided evidence of OS innervation within and surrounding the tumour in association with mesenchymal stroma that also corresponded to the most proliferative area of the tumour (Ki-67+). In vitro, both MSC and, to a lesser extent, OS cells promoted axonal growth through cytokine (IL-6) and neuromodulator (BDNF) secretion. Extracellular acidosis – a hallmark of OS aggressiveness – amplified IL-6 release by stromal cells, and its pro-neurogenic effect was prevented by IL-6 blockade. In turn, tumour-associated innervation stimulated OS cell proliferation and migration, eventually driving tumour aggressiveness.

**Conclusions:**

We showed, for the first time, that bone-associated nerves, fostered by the OS microenvironment, promote tumour aggressiveness. Interfering with the nerve-tumour axis, particularly with the signalling associated with mesenchymal stroma, offers novel opportunities for OS treatment.

**Supplementary Information:**

The online version contains supplementary material available at 10.1186/s13046-025-03532-2.

## Background

Osteosarcoma (OS) is the most common primary malignancy of bone in adolescents and young adults and a leading cause of cancer-related mortality in this population [1]. Recent advances have shown the pivotal role of tumour microenvironment (TME) in OS progression, opening novel therapeutic opportunities [2–4]. Among the several components of TME, nerves have been extensively investigated in carcinomas, and their presence within and surrounding the tumour has been correlated with prognosis [5–11]. While neural signalling in OS has also been reported [12–15], the interplay between neurons and OS cells and the role of innervation in disease progression have not been addressed so far.

Similar to and alongside angiogenesis, *de novo* formation of nerve fibres is a consistent feature of most malignancies. Tumours recruit nerves and stimulate nerve outgrowth (tumour axonogenesis) via the secretion of axon guidance molecules, such as neurotrophins (e.g. nerve growth factor, NGF; brain-derived neurotrophic factor, BDNF) and exosomes [16, 17]. Conversely, nerve-derived neurotransmitters and neuropeptides may promote immune evasion, tumour angiogenesis, and metastasis [18–20]. Besides, perineural invasion – a pathway for cancer cell dissemination along peripheral nerve sheaths – can further enhance cancer progression [21].

Within the Haversian canals in the cortical bone, and in central and peripheral marrow regions [22, 23], bone harbours intricate adrenergic and sensory neural networks that regulate tissue homeostasis and metabolism under physiological conditions [24–27] and likely represent the substrate for nerve-tumour crosstalk in OS. Previous studies in bone cancers, including OS, multiple myeloma, and bone metastases, have shown adrenergic signalling in the tumour tissue and cancer cell lines [12, 28–33]. Pain is also a common feature of malignancies arising in bone and negatively affects the prognosis of bone metastases [34–36]. Nevertheless, the mechanisms underlying the role of sensory nerves in the progression of bone cancers remain largely unexplored.

Our study addresses a critical gap in understanding the role of tumour-driven innervation in OS. We showed that tumour-associated mesenchymal stroma, activated by cancer cells, significantly contributes to OS progression by fostering pro-tumorigenic features of bone-associated nerves. Specifically, this occurs through the induction of both neurotrophic [37, 38] and indirect pro-tumorigenic phenotypes of mesenchymal stromal cells (MSC) [3, 4, 39, 40]. These findings highlight the pivotal role of the cancer-stromal-nerve crosstalk in OS pathophysiology, offering novel therapeutic opportunities.

## Methods

### Clinical study

Medical records of pet dogs diagnosed with OS at the Veterinary Hospital of the University of Bologna (Italy) between January 2015 and December 2018 were retrospectively analysed. Owners gave informed consent to the use of clinical data and stored biological samples for teaching and research purposes. Inclusion criteria required the availability of pre-chemotherapy tissue samples (*n* = 11).

For the sampling of human OS tissues, we retrospectively enrolled patients (*n* = 5) of both sexes, with recurrent OS, and previously treated at the IRCCS Rizzoli Orthopaedic Institute. The patients had not received chemotherapy in the year before surgery and had been subjected to amputation. The clinical study was approved by the Ethics Committee (local Ethics Committee, approval No. 184, 07/01/2015). All participants provided written informed consent.

For the sampling of not-transformed bone tissue, we prospectively enrolled patients (*n* = 3) of both sexes (age ≥ 65 years), subjected to hip replacement surgery for end-stage osteoarthritis or admitted to the emergency unit of the IRCCS Rizzoli Orthopaedic Institute for a fragility fracture of the femoral neck. The clinical study was approved by the Ethics Committee (CE-AVEC 586/2020/Sper/IOR, approval No. 0009411, 06/07/2020). All participants provided written informed consent.

For all types of samples, formalin-fixed tissues were paraffin embedded following decalcification (when necessary). Sections (5 μm thick) were stained with haematoxylin and eosin for histologic evaluation. For βIII-tubulin and Ki-67 immunohistochemistry, serial sections from representative blocks were prepared. Briefly, tissue sections mounted on silane-coated slices underwent dewaxing in Citro Histoclear (Histo-Line Laboratories, Milano, Italy), rehydration through graded ethanol, antigen retrieval (for Ki-67), endogenous peroxidase quenching (3% hydrogen peroxide), and blocking with 2% bovine serum albumin (BSA). Full details of βIII-tubulin and Ki-67 staining and relative quantification protocols are provided in the Supplementary Information section.

### Cell cultures

Human bone marrow-derived MSC were purchased from Lonza (Basel, Switzerland) and cultured in Minimum Essential Medium Eagle Alpha Modified (Alpha-MEM) (Sigma-Aldrich, St. Louis, MO, USA). Cancer-associated fibroblasts (CAF) were isolated from a human OS tissue sample following ethical approval (n. 20204 of 31.07.09) and informed consent. The sample was mechanically dissociated, and the resulting cell suspension cultured in Iscove’s Modified Dulbecco’s Medium (IMDM) (Life Technologies, Carlsbad, CA, USA) supplemented with 20% fetal bovine serum (FBS). Both MSC and CAF were used between passages 5–6. The human OS cell lines HOS and 143B (ATCC, Manassas, VA, USA) were cultured in IMDM. Cell line authentication was performed within three years via SNP profiling (LGC Standard, Teddington, UK). All the cells were cultured in the specified medium with 100 U/mL penicillin, 100 mg/mL streptomycin, and 10% FBS (Euroclone, Milan, Italy) (complete medium), under standard conditions (37 °C, 5% CO_2_, humidified atmosphere).

Rat neonatal sensory dorsal root ganglion (DRG) neurons (Lonza, Basel, Switzerland) were seeded on poly-D-lysine (0.1 mg/mL) and laminin (5 µg/mL)-coated supports (Sigma-Aldrich). Neurons were cultured in Primary Neuron Basal Medium (Lonza) supplemented with 2 mM L-glutamine, 50 µg/mL gentamicin, 37 ng/mL amphotericin, and 2% neural cell-survival factor (NSF-1, Lonza) (complete Neuron Basal medium, cNBM), under standard conditions.

pH-adjusted media were prepared by modulating sodium bicarbonate concentration according to the Henderson–Hasselbach equation [41]. Acidic and physiological media were buffered to pH 6.8 and pH 7.4, respectively, with pH levels verified throughout experiments.

Cell culture supernatants (conditioned media, CM) were collected as described in Table 1 and Supplementary Fig. S1 and stored at − 80 °C until use. Protocol for MSC conditioning, with OS cell supernatant, is detailed in the Supplementary Information section.

**Table 1:** Protocols for obtaining the different conditioned media (CM) used in the experimental assays to evaluate the effects of cell secretome

Abbreviation	Cell source	Type of support	*N*. of cells	Pre-treatments	Medium	Time of collection	Collection procedure
Neuron CM	DRG neurons	24-well precoated plates	2.5 × 10^3^/well	None	Complete Neuron Basal medium (cNBM)	48 h	Centrifugation at 1800 rpm (5 min at 4 °C)
MSC CM	MSC	T75 flask	70% confluence	Wash with PBS	cNBM w/o NSF-1	48 h	Centrifugation at 6000 rpm (15 min at 4 °C), with Vivaspin 20 (10 kDa MWCO; GE Healthcare, Chicago, IL, USA)
CAF CM	CAF	T75 flask	70% confluence	Wash with PBS	cNBM w/o NSF-1	48 h	Centrifugation at 6000 rpm (15 min at 4 °C), with Vivaspin 20 (10 kDa MWCO)
HOS CM	HOS	T75 flask	70% confluence	Wash with PBS	cNBM w/o NSF-1	48 h	Centrifugation at 6000 rpm (15 min at 4 °C), with Vivaspin 20 (10 kDa MWCO)
143B CM	143B	T75 flask	70% confluence	Wash with PBS	cNBM w/o NSF-1	48 h	Centrifugation at 6000 rpm (15 min at 4 °C), with Vivaspin 20 (10 kDa MWCO)
MSC^pH6.8^ CM	MSC	T25 flask	3 × 10^5^	After cell adhesion, incubation for 10 h with Alpha-MEM 0.1% FBS (low-serum medium); wash with PBS; incubation for 24 h with low-serum medium at pH 6.8; wash with PBS	cNBM w/o NSF-1	48 h	Centrifugation at 6000 rpm (15 min at 4 °C), with Vivaspin 20 (10 kDa MWCO)
MSC^pH7.4^ CM	MSC	T25 flask	3 × 10^5^	After cell adhesion, incubation for 10 h with Alpha-MEM 0.1% FBS (low-serum medium); wash with PBS; incubation for 24 h with low-serum medium at pH 7.4; wash with PBS	cNBM w/o NSF-1	48 h	Centrifugation at 6000 rpm (15 min at 4 °C), with Vivaspin 20 (10 kDa MWCO)
Pre-treated neuron CM	DRG neurons	24-well pre-coated plates	2.5 × 10^3^/well	At 48 h after cell seeding, wash with PBS, and incubation for 48 h with MSC^pH6.8^ CM or MSC^pH7.4^ CM; wash with PBS	cNBM w/o NSF-1	48 h	Centrifugation at 1800 rpm (5 min at 4 °C)

### Cell-cell contact quantification

MSC (1 × 10^2^ cells/well) and 143B (4 × 10^2^ cells/well) were seeded onto pre-coated 8-well chamber slides in complete α-MEM or complete IMDM, respectively. After 24 h under standard conditions, cells were washed, and the medium was replaced with cNBM containing DRG neurons (1.75 × 10^3^ cells/well). Following 72 h of co-culturing, cell-cell contacts were quantified under bright-field microscopy and normalized to the MSC/143B cell count. For image analysis and quantification, cell distinction relied on morphological characteristics, through the direct observation at the optical microscope. Neurons exhibit distinctive pseudounipolar morphology with neuritic processes, whereas OS or MSC cells appear as flat, mesenchymal-like cells with higher nuclear-to-cytoplasmic ratios.

For full details of the βIII-tubulin immunofluorescence staining protocol, refer to the Supplementary Information. Data were collected from three independent biological experiments.

### Neuron culture in microfluidic devices

To assess the effects of CM on axonal recruitment, polydimethylsiloxane (PDMS) AXIS™ Axon Isolation microfluidic devices (AX150, Sigma-Aldrich) were used. These include a somal compartment for neuron seeding and an axonal compartment for CM (Supplementary Fig. S2). Microfluidic devices were prepared as previously described [42]. For device sterilization, coverslips (24 × 24 mm) were treated with 1 M HCl (24 h shaking at 37 °C), rinsed with 96% ethanol, and stored in 70% ethanol o.n. AXIS devices were sterilized with 70% ethanol. For device assembly, dried devices were mounted onto dried coverslips, placed in 6-wells plates, coated with 0.1 mg/mL poly-D-lysine, washed with water, and incubated with 5 µg/mL laminin o.n. at 37 °C and in a humidified 5% CO2 atmosphere. For neuron seeding, chambers were then filled with cNBM and neurons (2.5 × 10^4^ cells) were added to each somal compartment channel. Hydrostatic flow was induced by adding 200 µL and 100 µL cNBM to the somal and axonal compartments, respectively. Medium was replaced every 72 h. After 6 days, axons were observed at the somal-axonal interface. CM (Table 1) or control medium (cNBM w/o NSF-1, not exposed to cells; CTR) were added to the axonal compartment. Neurons were cultured for additional 6 days (medium refreshed every 72 h). Axon-filled channels and axonal growth were quantified. Experiments were performed in biological duplicate with at least two technical replicates per experiment.

### Axonal growth quantification

#### Static cultures

Neurons (7.5 × 10^3^ cells/well) were seeded onto pre-coated chamber slides. After adhesion, cells were washed and incubated for 72 h with: (1) CM (Table 1 and Supplementary Information for MSC ^143B CM^ CM); (2) control medium (cNBM w/o NSF-1 not exposed to cells, CTR); or (3) positive control (50 ng/mL NGF, Sigma-Aldrich). In a subset of experiments, neurons were immunostained for βIII-tubulin by immunofluorescence to visualize axons (Supplementary Information). For interleukin-6 (IL-6) blocking experiments, neurons were pre-treated with 100 µg/ml anti-IL6 receptor antibody (tocilizumab, TCZ) for 48 h prior to CM exposure, with treatments replenished every 24 h. For BDNF blocking experiments, neurons were pre-treated with 1 µg/ml anti-BDNF receptor antibody (Anti-BDNF Ab) (PeproTech, Cranbury, NJ, USA) for 48 h prior to CM exposure, with treatments replenished every 24 h. For IL-6 treatment experiments, neurons were exposed to 10 and 25 ng/mL IL-6 Recombinant Protein (PeproTech) for 72 h, with treatments replenished every other day. For BDNF treatment experiments, neurons were exposed to 20 and 50 ng/mL BDNF Recombinant Protein (PeproTech) for 72 h, with treatments replenished every other day.

#### Microfluidic cultures

Neurons were seeded into AXIS™ Axon isolation devices and cultured for 6 days (as described above). CM was then added to the axonal compartment, and after additional 6 days, axon length was quantified using ImageJ software. The five longest axons were considered to determine the maximum average length per device.

### ELISA

Pro-neurogenic mediators in CM were quantified using the following kits: Human IL-6 and Free BDNF Quantikine ELISA Kits (R&D Systems, Minneapolis, MN, USA) and Human beta-NGF ELISA Kit (Thermo Fisher Scientific, Waltham, MA, USA). Protein concentrations were normalized to total protein content measured by bicinchoninic acid (BCA) assay. Assays were performed in duplicate across two independent experiments.

### Cell counting and viability assays

#### OS cell-neuron co-cultures

Human OS cell lines 143B (1.8 × 10^3^ cells/well) and HOS (2 × 10^3^ cells/well) were seeded into pre-coated 48-well plates in complete IMDM. After 7 h, the medium was replaced with either: (1) cNBM containing neurons (1.8 × 10^3^ cells/well; co-culture); (2) cNBM alone (OS monoculture, negative control). Both co-culture and monoculture media were supplemented with 2% NSF-1. After 96 h, cells were stained with 1 µg/mL DAPI in IMDM. Automated imaging was performed using the ImageXpress Pico system (4x lens, Molecular Devices, San Jose, CA, USA), and nuclei were quantified by CellReporterXpress software. OS cells were distinguished from neurons based on nuclear diameter (OS: 11–14 μm; neurons: 3–10 μm). Assays were made in triplicate across two independent experiments.

#### OS exposed to neuron CM

143B (9 × 10^2^ cells/well) and HOS (1 × 10^3^ cells/well) cells were seeded into 96-well plates in complete IMDM. After 48 h, medium was replaced with neuron CM (prepared as per Table 1) for an additional 48 h. Nuclei were DAPI-stained (1 µg/mL) and quantified as above. For Alamar blue assay, cells were incubated with 10% Alamar Blue (Invitrogen, Waltham, MA, USA) for 4 h, and fluorescence measured at 540/590 nm (excitation/emission) using a Tecan Infinite F200pro plate reader, following manufacturer guidelines. Assays were performed in quadruplicate across two experiments.

#### OS exposed to pre-treated neuron CM

Neurons were pre-treated as outlined in Table 1. For TCZ experiments, neurons were additionally exposed to 100 µg/mL TCZ 48 h prior to MSC^pH6.8^ CM treatment. 143B (9 × 10^2^ cells/well) were seeded into 96-well plates in complete IMDM. After 48 h, cells were incubated with pre-treated neuron CM for additional 48 h. Viability was assessed via Alamar blue assay, as described. Assays were conducted in quadruplicate across two independent experiments.

### Migration assays

#### Direct co-culture

Protocols for migration assays in direct neuron-OS co-cultures are detailed in the Supplementary Information.

#### Transwell migration assay

Neurons (1.5 × 10^4^ cells) were seeded in pre-coated 24-well plates and cultured in cNBM. Parallel wells containing cNBM without neurons served as a negative control. After 48 h, 143B and HOS cells (3 × 10^4^) were seeded into 8 µm-pore transwell inserts, which were transferred to wells containing either pre-incubated neuron-free cNBM medium (- neurons) or pre-cultured neurons (+ neurons). Both the ‘- neurons’ control and the ‘+ neurons’ conditions were supplemented with 2% NSF-1. Following 72 h for 143B or 48 h for HOS, migrated cells were fixed in methanol, stained with crystal violet, and counted across nine random fields (20× lens). Assays were performed in triplicate across two independent experiments.

### Statistical analysis

Data were analysed using GraphPad Prism 7.0 (GraphPad Software, San Diego, CA, USA) and expressed as mean ± standard error of the mean (SEM). Given the limited sample size, non-parametric tests were used. For group comparisons, we used the Mann-Whitney U test. To distinguish the type of replicates, we named intra-assay the technical replicates, and inter-assay the independent biological replicates. To assess the correlation between the number of βIII-tubulin-positive nerve fibres and the Ki-67 proliferation index in canine OS tissues, Spearman’s rank correlation analysis was performed. For all the analyses, a *p* ≤ 0.05 was considered statistically significant.

## Results

### Nerve infiltration in OS-associated stroma

To assess the presence of nerves in tumour tissue, we performed βIII-tubulin immunostaining in a retrospective series of canine and human OS samples (*n* = 11 and *n* = 5, respectively). Clinical and histological features are listed in Tables 2 and 3, respectively.

**Table 2:** Clinical and histological features of canine OS

Patient	Gender	Age	Breed	Histological subtype	Site	Innervation index based on βIII-tubulin expression	Ki-67 index (%, mean ± SE)
1	F	7	Leonberger	High-grade osteoblastic	Tibia	None	0
2	M	12	Rottweiler	High-grade osteoblastic, fibroblastic, and chondroblastic	Tibia	+	37.23 ± 1.27
3	M	9	Rottweiler	High-grade osteoblastic	Femur	++	43.56 ± 4.78
4	M	11	Rottweiler	High-grade osteoblastic	Humerus	+	15.98 ± 0.94
5	F	9	Half-breed	High-grade osteoblastic	Femur	+++	62.00 ± 1.71
6	M	4	Half-breed	High-grade osteoblastic	Tibia	++	43.14 ± 3.37
7	M	7	Maremma-Abruzzese Shepherd	High-grade fibroblastic	Femur	++	41.25 ± 1.60
8	F	4	Half-breed	High-grade osteoblastic	Humerus	+	35.33 ± 1.57
9	F	13	Half-breed	High-grade fibroblastic and chondroblastic	Radius	+++	70.50 ± 3.53
10	M	9	Czechoslovakian Wolfdog	High-grade osteoblastic	Radius	+++	87.43 ± 1.90
11	F	9	Half-breed	High-grade osteoblastic	Tibia	++	76.06 ± 4.18

**Table 3:** Clinical and histological features of human OS

Patient	Gender	Age	Histological subtype	Site	Innervation index based on βIII-tubulin expression
OS1	F	19	Recurrent high-grade osteoblastic and fibroblastic	Tibia	+++
OS2	F	55	Recurrent high-grade osteoblastic and fibroblastic	Humerus	+++
OS3	F	68	Recurrent high-grade osteoblastic and fibroblastic	Hip	++
OS4	F	22	Recurrent high-grade osteoblastic and fibroblastic	Femur	++
OS5	F	23	Recurrent high-grade osteoblastic	Gluteus	+++

**Fig. 1:**
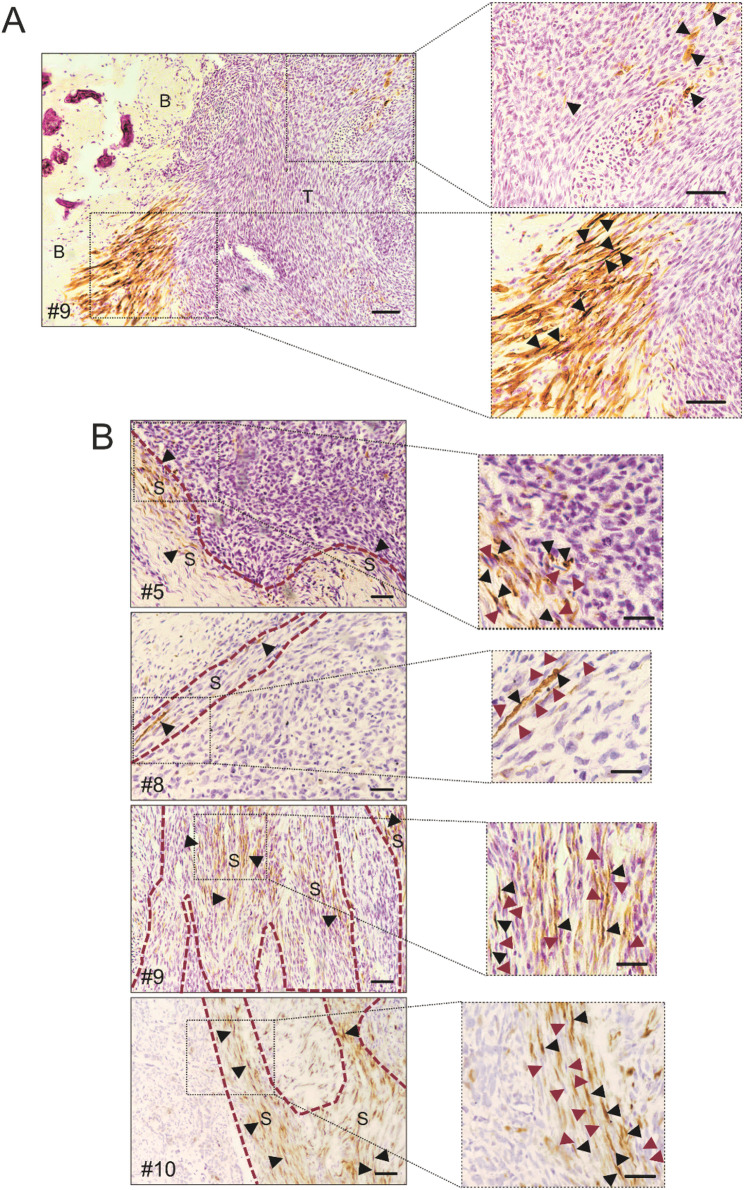
Nerve fibres in canine OS tissues. βIII-tubulin immunohistochemistry in spontaneous canine OS. (**A**) Representative image of βIII-tubulin + nerve fibres (axons). High-magnification insets show fibres (arrows) infiltrating tumour parenchyma and at the bone-tumour interface (B, bone; T, tumour). Scale bar = 100 μm. (**B**) βIII-tubulin + nerve fibres (black arrows) localized within stromal regions (S, stroma) across four representative OS cases. High-magnification insets highlight fibres (black arrows) adjacent to tumour-associated stromal cells (red arrows). Scale bar = 25 μm

**Fig. 2:**
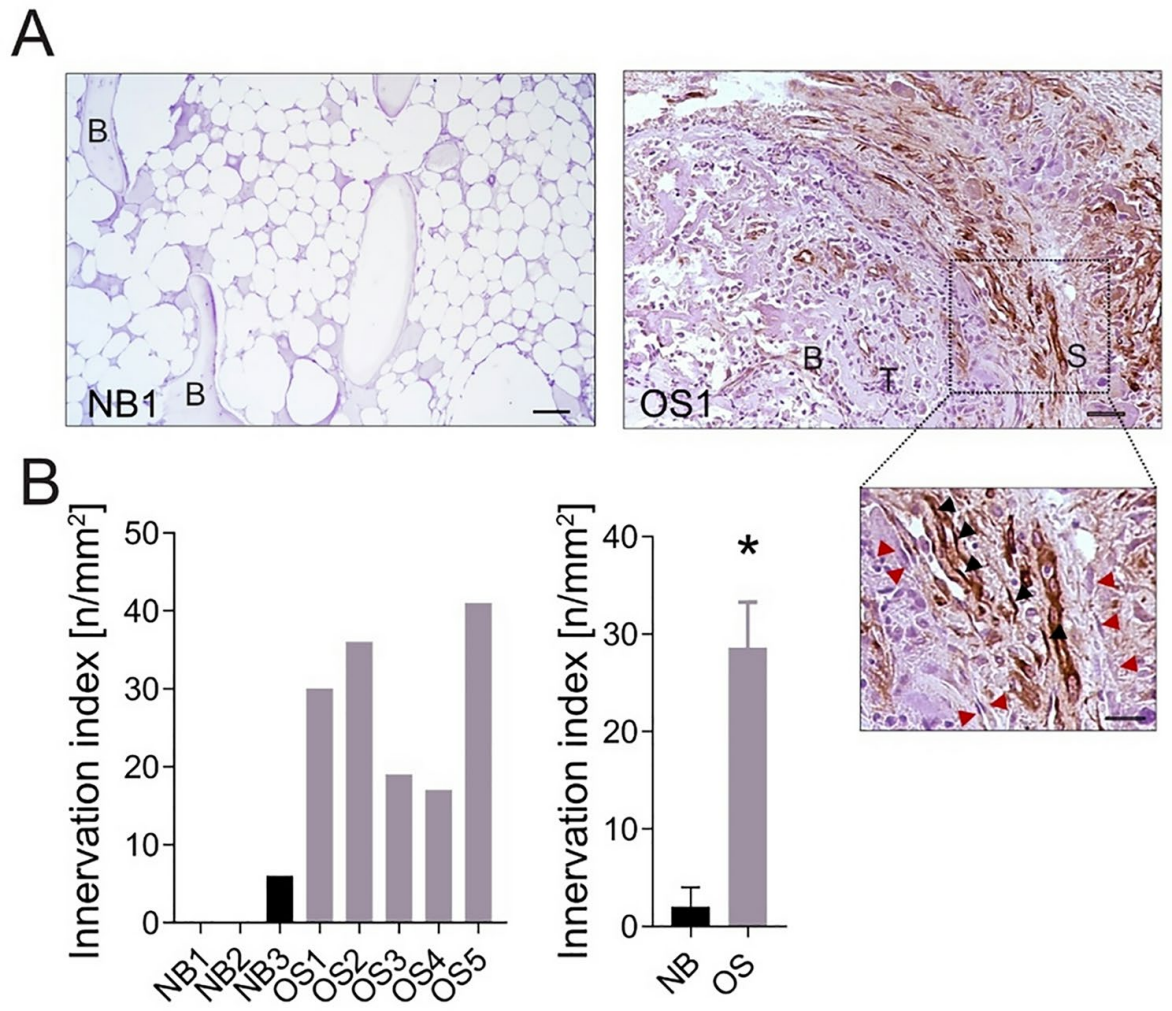
Innervation of human OS tissues vs. normal bones. (**A**) βIII-tubulin immunostaining in a representative normal human bone (NB1) and OS tissue (OS1), in the pseudocapsule region (infiltrating stroma, S; tumour, T, bone, B). High-magnification inset shows βIII-tubulin + axons (black arrows) adjacent to tumour-associated stromal cells (red arrows). Scale bar = 50 μm. **(B)** Quantification of nerve density in normal bones and OS tissues. Data are expressed as the number of βIII-tubulin + nerve fibres per area (innervation index, n/mm²). Left panel, single values; right panel, grouped values per category (*N* = 3 for NB, and *N* = 5 for OS, mean ± SE, *p < 0.05)

Neuronal marker-positive elongated and anucleate structures — morphologically consistent with axons — were identified in 10 out of 11 cases of canine OS (Fig. 1 and Supplementary Fig. S3A). While these fibrils partially infiltrated the tumour, they predominantly surrounded its periphery (arrows in Fig. 1A, Supplementary Fig. S4A, *p* = 0.0019), in close proximity to mesenchymal stroma (arrows in Fig. 1B, Supplementary Fig. S4B, *p* < 0.0001). We observed a similar spatial association between nerves and mesenchymal stroma in human OS tissues (Fig. 2A and Supplementary Fig. S5B, red arrows indicating tumour-associated stromal cells). βIII-tubulin-positive nerve fibres were detected in all human OS samples. In some cases, βIII-tubulin signal was also observed in the cytoplasm of stromal and tumour cells (Supplementary Fig. S5B, blue arrows). Importantly, nerve density was significantly higher in these human OS samples compared to normal human bone tissues, as shown by representative images (Fig. 2A and Supplementary Fig. S5B), and demonstrated by relative quantification (Fig. 2B, *p* = 0.0357). To validate the specificity of the anti-βIII-tubulin antibody, we used both negative and positive controls (Supplementary Fig. S6).

**Fig. 3:**
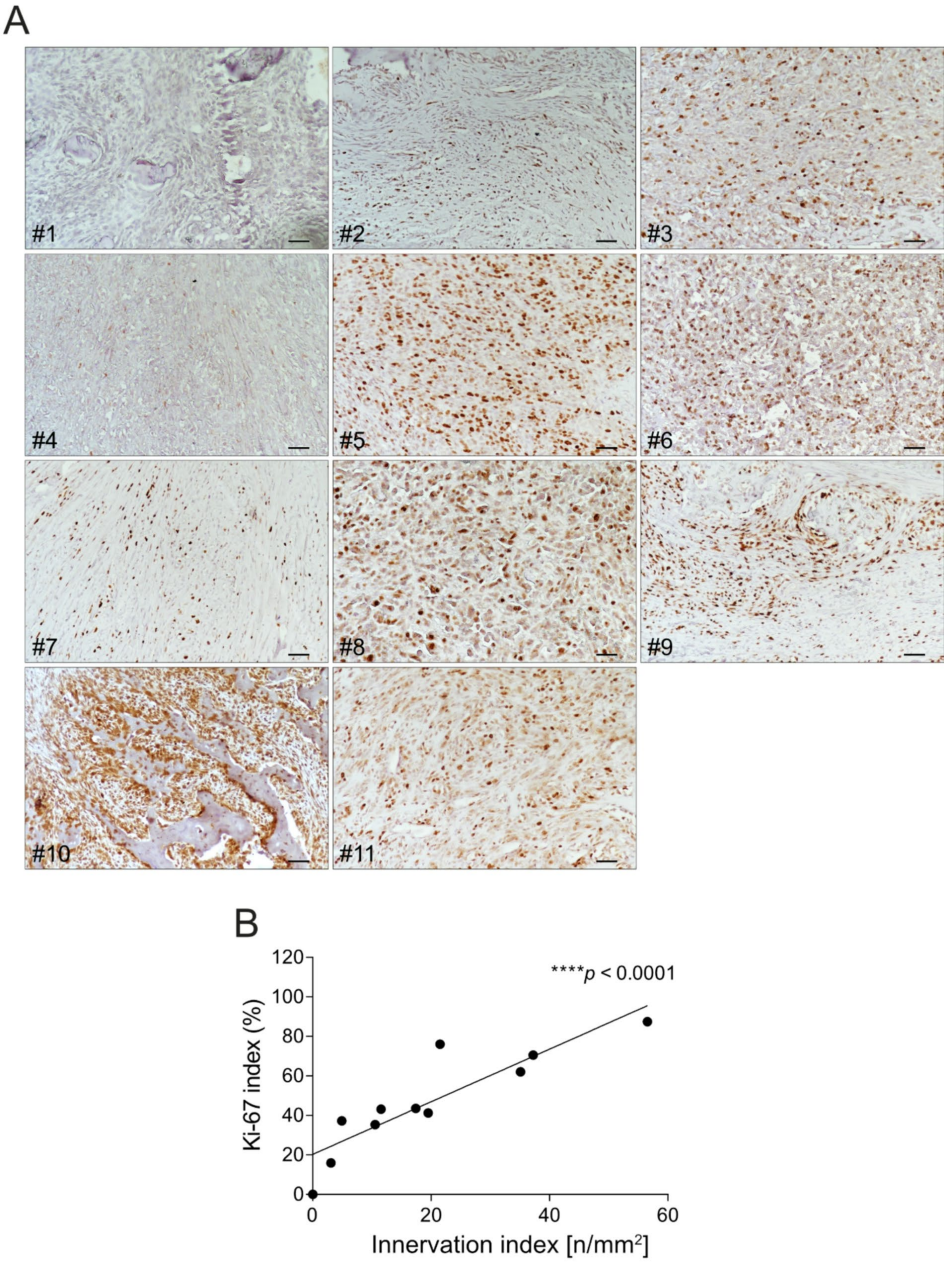
Correlation between nerve density and Ki-67 proliferation index in canine OS. (**A**) Representative Ki-67 immunohistochemistry images of canine OS tissue. Scale bar = 50 μm. (**B**) Correlation between Ki-67 proliferation index (% Ki-67 + cells among total tumour cells) and innervation index based on βIII-tubulin staining (mean ± SE, *N* = 11, ****p < 0.0001)

Finally, we found a strong positive correlation between the Ki-67 proliferation index and nerve fibre density in canine OS tissues (Fig. 3, *p* < 0.0001). This further supports a link between innervation and tumour aggressiveness.

In summary, histological analysis showed constant innervation within the stroma and the pseudocapsule of both canine and human OS tissues. The significant correlation with tumour cell proliferation index strongly suggests a potential interaction between the nervous system and OS microenvironment supporting tumour growth.

### Neurons exhibit greater tropism toward MSC

**Fig. 4:**
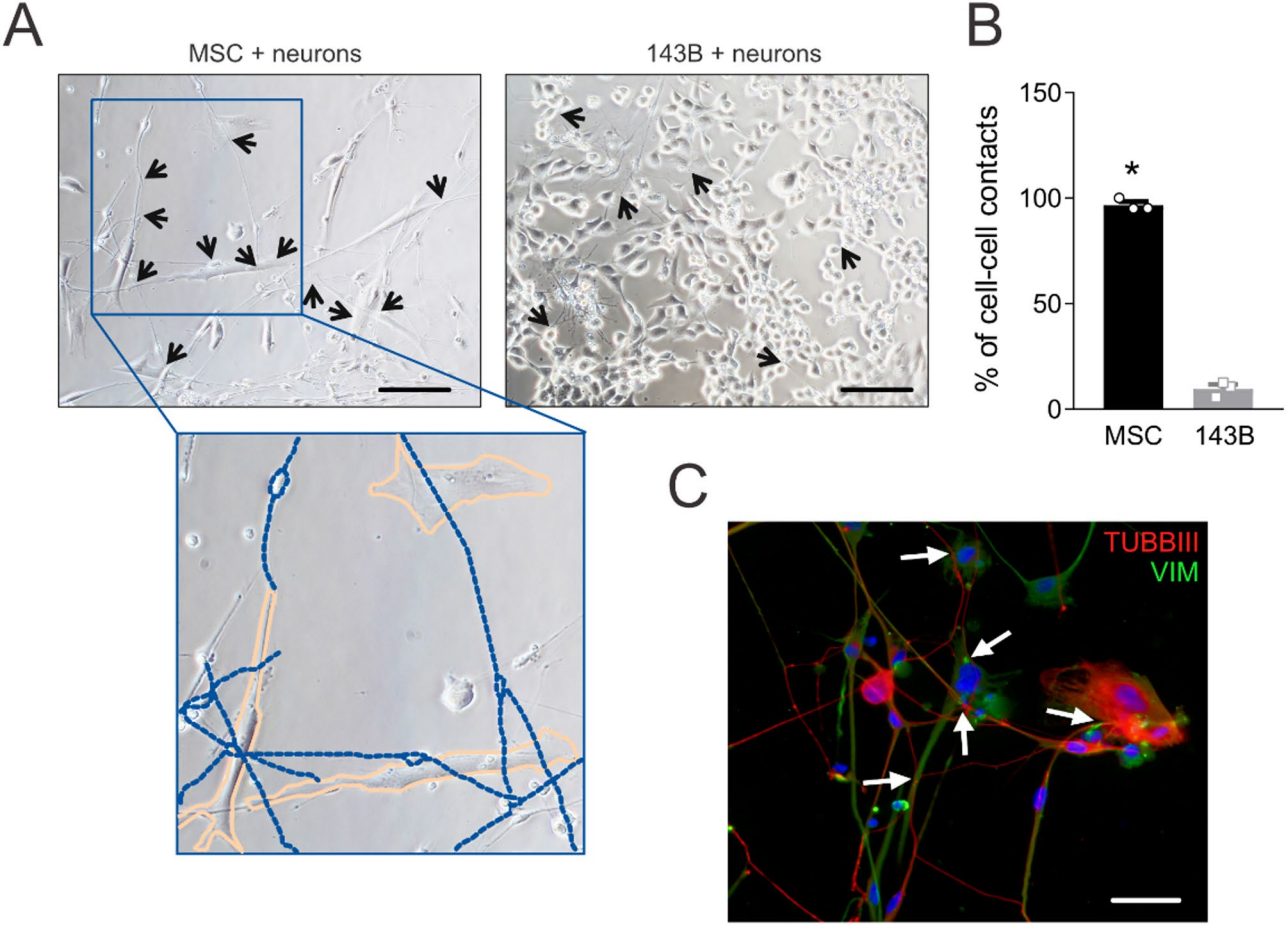
Mesenchymal stromal cells promote interactions with neurons more than OS cells. (**A**) Bright-field images of neurons co-cultured with MSC or 143B OS cells for 72 h. Increased neuron-MSC contacts (arrows) vs. neuron-143B co-cultures. Inset: neurons (blue dotted line) extended branched neurites around MSC (orange line), displaying pseudounipolar morphology (20×, scale bar 100 μm). (**B**) Quantification of neuron-MSC/143B contacts in the experiment shown in panel (a), normalized to cell number (mean ± SE, 3 inter-assay and 1 intra-assay replicates, *N* = 3, *p < 0.05). (**C**) Immunofluorescence of neurons-MSC co-culture: βIII-tubulin + neurites (red) extend toward vimentin + MSC (green, arrows) (40×, scale bar 50 μm)

Given the histological proximity of nerves to tumour-associated mesenchymal stroma, we assessed whether MSC promote axonal recruitment. In vitro, neurons formed significantly more contacts with MSC than with 143B OS cells (Fig. 4A, arrows; quantified in Fig. 4B, *p* = 0.0495). Immunofluorescence revealed βIII-tubulin-positive axons extending toward vimentin-positive MSC (Fig. 4C, arrows), forming branched networks upon contact (a representative image of co-culture between neurons and 143B cells is shown in Supplementary Fig. S7). Notably, neurons in neuron-MSC co-cultures retained the pseudounipolar morphology characteristic of DRG neurons (Fig. 4A, blue dotted lines in the inset). These findings suggest that tumour-associated mesenchymal stroma may actively facilitate the formation of a neuronal network within the TME of OS.

### Mesenchymal stroma enhances axon recruitment and elongation in a microfluidic model

To further compare the neurotrophic potential of OS-associated stromal cells and OS cells, we used CAF isolated from human OS tissue and MSC as in vitro models. CAF were identified by a limited proliferative capacity, reduced lifespan, and high expression of CAF markers (smooth muscle alpha (α)-2 actin, ACTA2, and Fibroblast Activation Protein Alpha, FAP, Supplementary Fig. S8, *p* = 0.0286) [43].

**Fig. 5:**
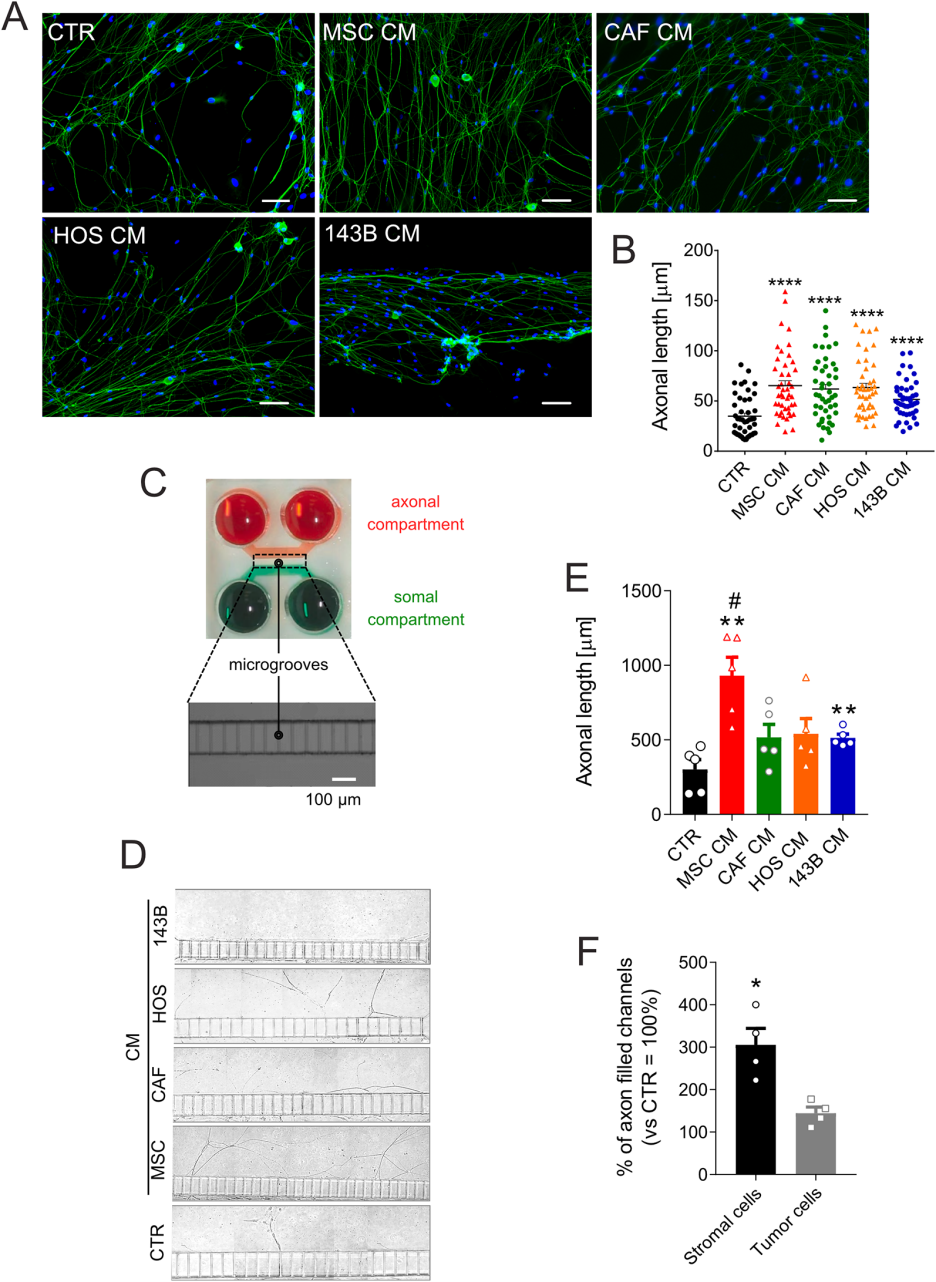
Stromal and OS cell-conditioned medium promote axonal outgrowth. (**A**) βIII-tubulin immunofluorescence (green) in DRG neurons exposed for 72 h to CM or control medium (CTR, neuron basal medium) (20× lens, scale bar 100 μm). Nuclei counterstained with Hoechst 33,258 (blue). (**B**) Quantification of axon length across conditions represented in panel (a) (mean ± SE, 3 inter-assay and 15 intra-assay replicates, *N* = 45, ****p < 0.0001 vs. CTR). (**C**) Schematic view of the microfluidic axon isolation device: somal (green) and axonal (red) compartments separated by microgrooves (150 μm length). (**D**) Bright-field images of axons crossing microgrooves into the axonal compartment. Neurons treated with CTR (cNBM), or CM. (**E**) Quantification of neurite length across conditions shown in panel (d) (mean ± SE, 2 inter-assay replicates and least 2–3 intra-assay replicates, *N* = 5, **p < 0.01 vs. CTR, ^#^*p* < 0.05 vs. 143B). (**F**) Quantification of axon-filled channels for stromal (MSC and CAF) vs. OS (HOS and 143B) cells relative to CTR (cNBM, = 100%) shown in panel (d) (mean ± SE, 2 inter-assay and 2 intra-assay replicates, *N* = 4, *p < 0.05, results are expressed as percentage)

CM from MSC and CAF significantly enhanced axonal outgrowth (Fig. 5A-B), with effects comparable to OS cell-derived CM (Fig. 5B, *p* = 0.0003 for MSC; *p* < 0.0001 for others). However, on average, stromal cells (MSC and CAF) induced longer neurites than OS cells (143B and HOS) (63.56 ± 3.32 μm vs. 57.39 ± 2.57 μm), although this difference was not statistically significant.

Using a microfluidic device for spatial and fluidic isolation of axons (Fig. 5C), we observed robust axon penetration into CM-containing chambers in all conditions. By image analysis, we found that MSC CM and 143B CM significantly promoted axon elongation (Fig. 5E, *p* = 0.0079 vs. CTR), with MSC exhibiting a greater effect (Fig. 5E, *p* = 0.0159 vs. 143B CM). Furthermore, by analysing the percentage of axon-filled channels, stromal cells were more effective than OS cells (Fig. 5F, *p* = 0.0286). We then aimed to assess whether axonal attraction toward MSC and 143B cells occurs in a setting that mimics in vivo conditions. To do this, we used a second microfluidic chip platform. This system enables the co-culture of heterotypic spheroids—composed of both MSC and 143B OS cells—embedded in Matrigel to mimic the extracellular matrix (ECM), as previously shown [39]. Neurons were seeded in adjacent lateral channels, spatially separated from the central chamber containing the embedded spheroid (Supplementary Fig. S9A and B). This microfluidic model recreates a 3D architecture similar to native tissue. It also supports the formation of natural gradients of metabolites and nutrients within the extracellular and intercellular spaces. Consistent with our previous findings, this assay showed strong axon outgrowth from the neuronal channels toward the MSC/143B spheroid embedded in the matrix (Supplementary Fig. S9C), further supporting a strong neurotrophic effect under these conditions. Finally, using a time-lapse co-culture assay (Supplementary Video V1) and a Transwell migration assay, we showed that the chemotactic interaction is bidirectional. Nerves can also attract MSC (Supplementary Fig. S10, *p* = 0.0195).

Overall, our findings confirmed that OS-associated mesenchymal stroma secretes factors that promote nerve recruitment and axon elongation at a higher extent than tumour cells.

### Stromal-derived BDNF and IL-6 drive tumour-associated axonal outgrowth

**Fig. 6:**
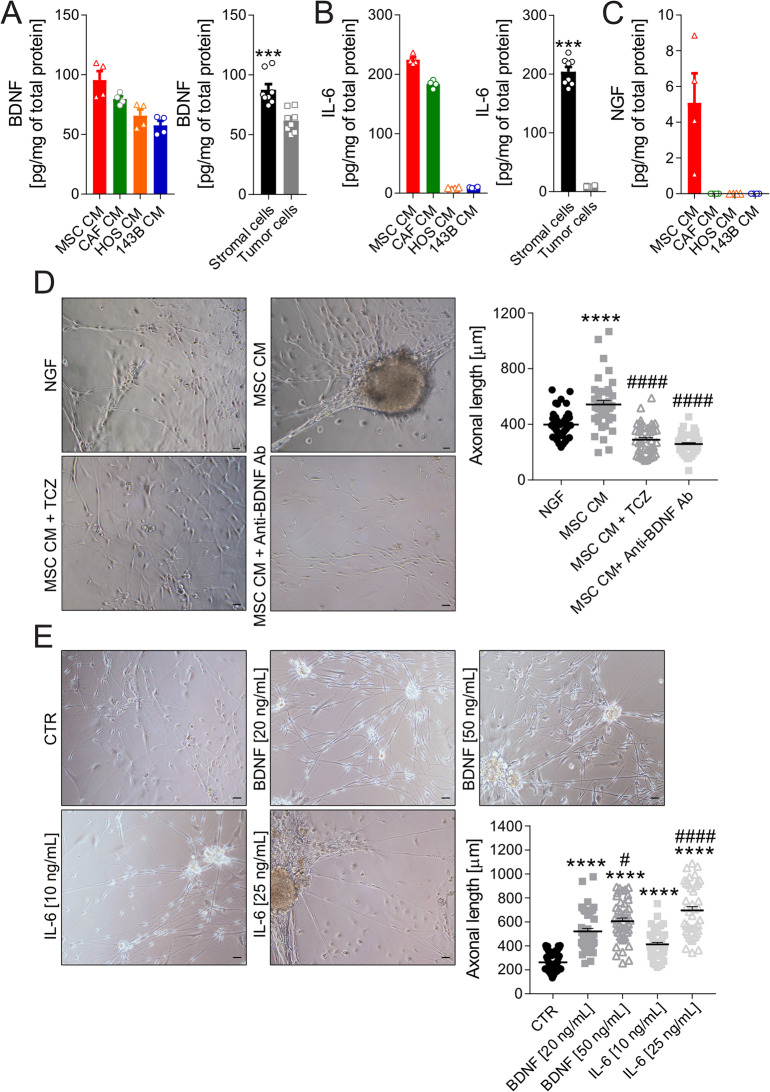
Stromal-derived BDNF and IL-6 drive axonogenesis. ELISA quantification of BDNF (**A**), IL-6 (**B**), and (**C**) NGF levels in CM from OS TME cells after 48-h incubation in neuronal basal medium. Left panel: absolute concentrations. Right panel: average for stromal (MSC and CAF) vs. OS (HOS and 143B) cell secretion ***p < 0.001 (mean ± SE, 2 inter-assay and 2 intra-assay replicates, *N* = 4) (**D**) DRG neurons exposed for 72 h to NGF (positive control), or MSC CM ± anti-IL6 antibody (Tocilizumab, TCZ), or MSC CM ± anti-BDNF antibody (Anti-BDNF Ab) (20× lens, scale bar 100 μm). On the right, the graph of axon length quantification (mean ± SE, 3 inter-assay and 15 intra-assay replicates, *N* = 45, ****p < 0.0001 vs. NGF, ^####^*p* < 0.0001 vs. MSC CM). (**E**) DRG neurons exposed for 72 h to increasing concentrations of BDNF and IL-6 (20× lens, scale bar 100 μm). On the right, the graph of axon length quantification (mean ± SE, 3 inter-assay and 15 intra-assay replicates, *N* = 45, ****p < 0.0001 vs. CTR (untreated cells), ^####^*p* < 0.0001 vs. IL-6 10 ng/mL, ^#^*p* < 0.05 vs. BDNF 20 ng/mL)

To identify neuromodulators driving nerve recruitment, we quantified NGF, BDNF, and IL-6 secretion by MSC, CAF, and OS cells (HOS and 143B) by ELISA. NGF was only detectable in MSC (5.09 ± 1.65 pg/mg total protein) (Fig. 6C), whereas BDNF was secreted by all cell types, with stromal cells exhibiting significantly higher levels than OS cells (Fig. 6A, *p* = 0.0003). IL-6 was predominantly released by stromal cells (Fig. 6B, *p* = 0.0002).

Blockage of IL-6 and BDNF with TCZ and a BDNF neutralizing antibody (anti-BDNF Ab), respectively, reduced MSC CM-induced axonal outgrowth (Fig. 6D, *p* < 0.0001). Notably, MSC CM stimulated axonal outgrowth more potently than exogenous NGF (Fig. 6D, *p* < 0.0001).

To further investigate the individual contributions of IL-6 and BDNF to axonogenesis, we evaluated their effects on axonal growth at two different concentrations. Both cytokines significantly promoted axon elongation in sensory neurons in a dose-dependent manner (Fig. 6E, *p* < 0.0001 vs. untreated cells). Notably, higher concentrations of IL-6 and BDNF induced significantly greater axonal extension compared to their respective lower doses, confirming a concentration-dependent effect (*p* < 0.0001 and *p* < 0.05, respectively).

**Fig. 7:**
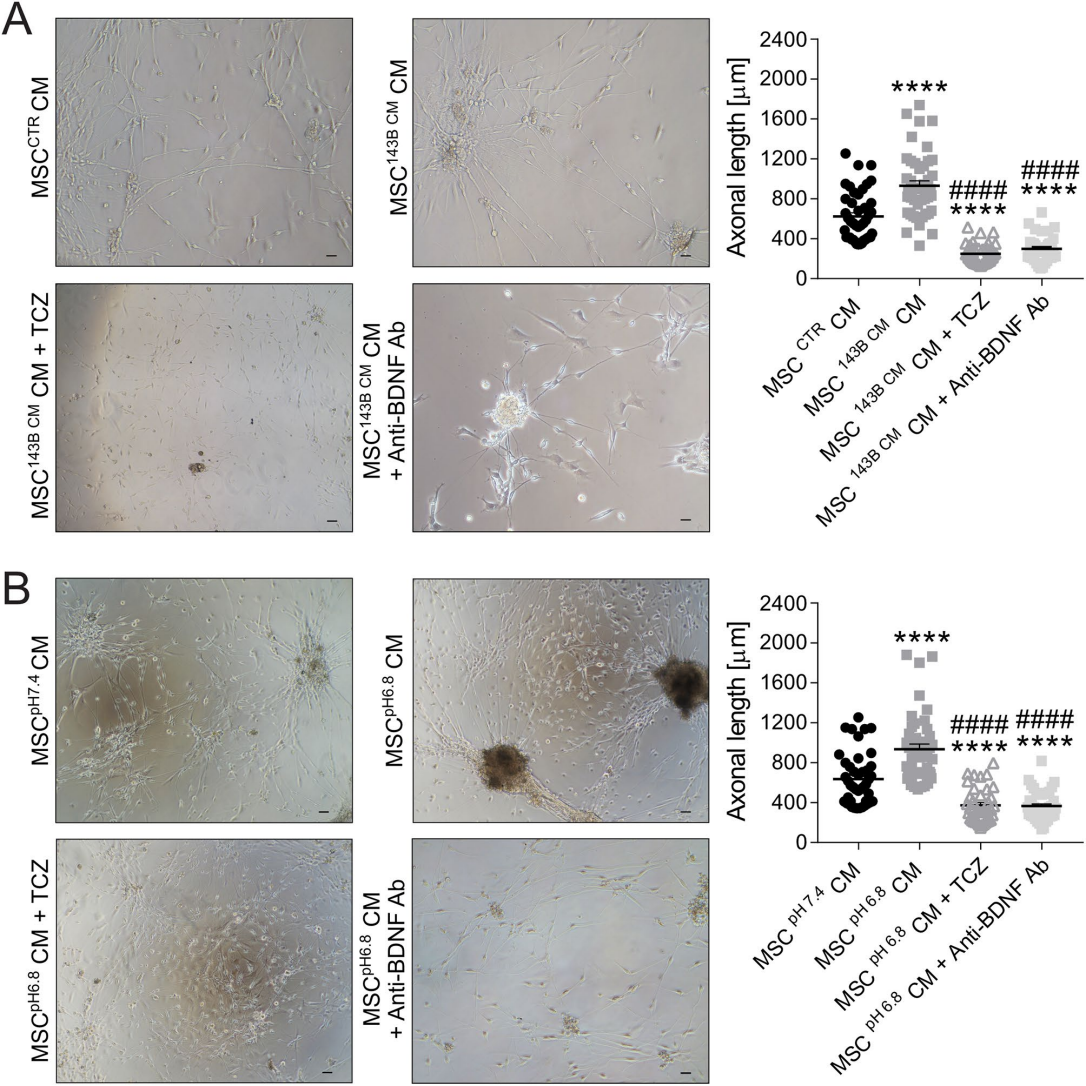
BDNF and IL-6 drive axonogenesis in the OS TME. (**A**) DRG neurons exposed for 72 h to the conditioned medium (CM) from MSC pre-incubated with the CM of 143B cells or control medium (MSC^CTR^ CM), ± the anti-IL6 antibody TCZ or anti-BDNF antibody (20×, scale bar 100 μm). The graph shows the relative quantification of axon length (mean ± SE, 3 inter-assay and 15 intra-assay replicates, N = 45, ****p < 0.0001 vs. MSC^CTR^ CM, ^####^*p* < 0.0001 vs. MSC^143B CM^ CM). (**B**) DRG neurons exposed for 72 h to CM from MSC pre-incubated in neutral (MSC^pH7.4^ CM) or acidic medium (MSC^pH6.8^ CM), ± the anti-IL6 antibody TCZ or anti-BDNF antibody (20×, scale bar 100 μm). The graph shows the relative quantification of axon length (mean ± SE, 3 inter-assay and 15 intra-assay replicates, N = 45, ****p < 0.0001 vs. MSC^pH7.4^ CM, ^####^*p* < 0.0001 vs. MSC^pH6.8^ CM)

To further confirm that MSC-derived IL-6 and BDNF are key mediators of axonogenesis in OS microenvironment, including when MSC are activated by tumour cells, we treated neurons with the supernatant from MSC pre-activated by exposure to 143B cell-conditioned medium (see experimental design in Supplementary Fig. S1). Treatment with the supernatant from tumour-activated MSC (MSC^143B CM^ CM) significantly promoted axonogenesis (Fig. 7A, *p* < 0.0001 vs. MSC^CTR^ CM). Notably, neutralization of IL-6 or BDNF using specific functional antibodies significantly reversed this effect (*p* < 0.0001 for both, vs. MSC^143B CM^ CM).

In addition, given the recognized role of acidosis in enhancing IL-6 and BDNF secretion by MSC and the established presence of extracellular acidosis in OS [44], we investigated whether acid-stressed MSC (MSC^pH6.8^) further promote axonal growth. Indeed, neurons exposed to MSC^pH6.8^ exhibited significantly longer axons than those treated with MSC^pH7.4^ (Fig. 7B, *p* < 0.0001). Crucially, both TCZ and anti-BDNF antibody pre-treatment abrogated such effect (Fig. 7B, *p* < 0.0001 for both, vs. MSC^pH6.8^ CM).

Finally, similar to MSC, we found that other components of the OS microen**vi**ronment ˗ specifically normal human osteoblasts or tumour-infiltrating monocytes ˗ can also promote axonal outgrowth, through mechanisms involving BDNF and IL-6 (Supplementary Figure S11).

In conclusion, our data demonstrate that IL-6 and BDNF are key mediators of stromal-driven axonal outgrowth in OS. This effect might not be exclusive to MSC but also may involve other cellular elements of the TME. Notably, tumour-derived alterations of the microenvironment that are not limited to the secretion of paracrine factors, like tumour-associated acidosis, further amplify the MSC neurotrophic activity.

### Sensory neurons promote OS proliferation and migration

**Fig. 8:**
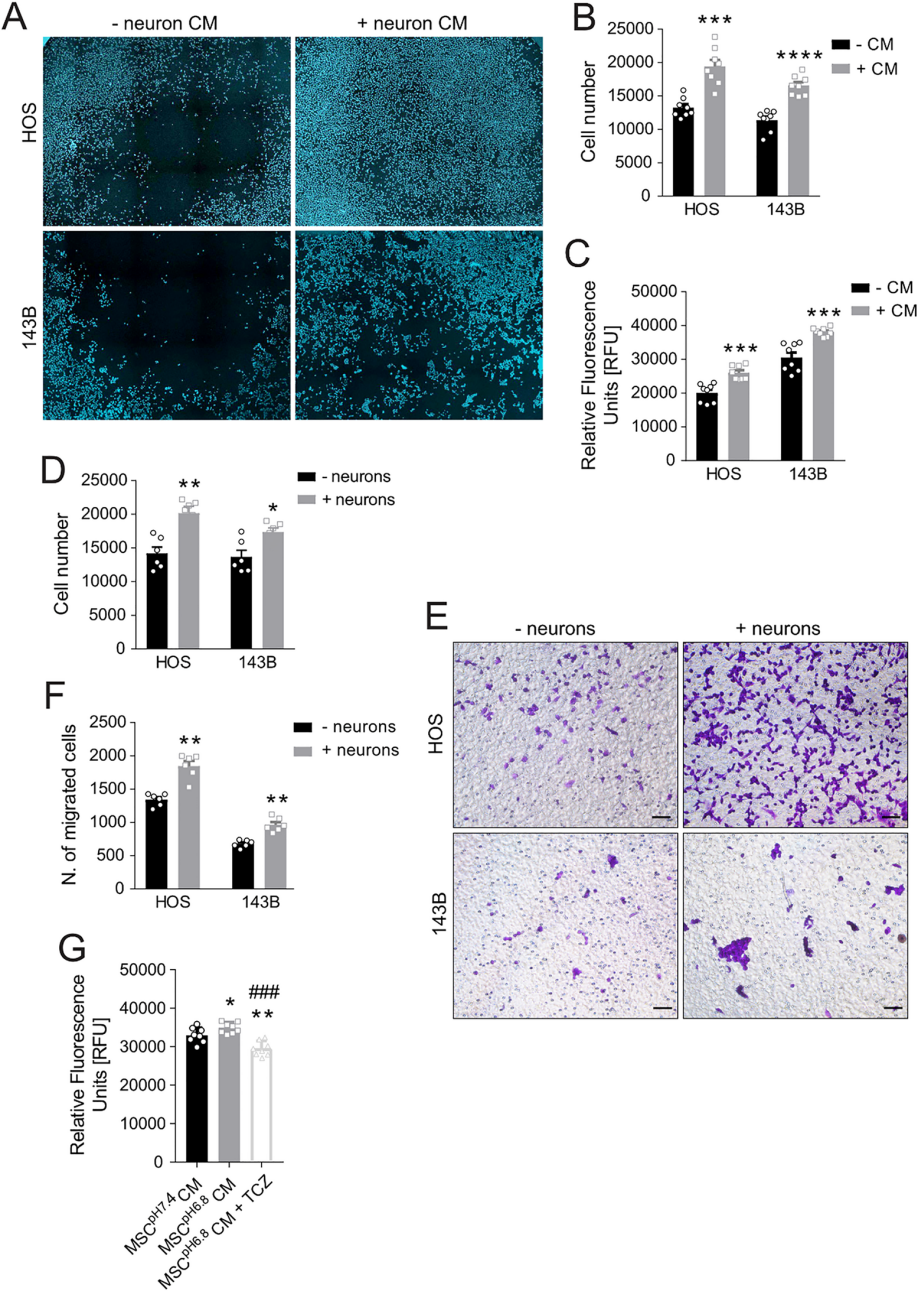
DRG neurons induce OS cell proliferation and migration.** (A)** Images of Hoechst 33,258-stained nuclei of HOS and 143B cells after 48-h exposure to neuron CM (4x, ImageXpress Pico) and **(B)** their automatic quantification (CellReporterXpress software, mean ± SE, 4 inter-assay and 2 intra-assay replicates, *N* = 8, ***p < 0.001 and ****p < 0.0001 vs. the respective -CM). **(C)** Alamar blue cell viability assay of HOS/143B cells after 48 h of exposure to neuron CM (mean ± SE, 4 inter-assay and 2 intra-assay replicates, *N* = 8, ***p < 0.001 vs. the respective -CM). **(D)** HOS/143B cell number after 48-h direct co-culture with neurons (mean ± SE, 3 inter-assay and 2 intra-assay replicates, *N* = 6, **p < 0.01 and *p < 0.05 vs. the respective -neurons). **(E)** Crystal violet-stained migrated HOS/143B cells on transwell membrane (20×, scale bar 100 μm). **(F)** Quantification of migrated cells (mean ± SE, 3 inter-assay and 2 intra-assay replicates, *N* = 6, **p < 0.01 vs. the respective -neurons). **(G)** Alamar blue viability assay of 143B OS cells 48 h after exposure to CM of neurons pre-treated with MSC^pH7.4^ CM or MSC^pH6.8^ CM for 48 h, ± TCZ. Relative Fluorescence Units (RFU) (mean ± SE, 4 inter-assay and 2 intra-assay replicates, *N* = 8, **p < 0.01, and *p < 0.05 vs. MSC^pH7.4^ CM, ^###^*p* < 0.001 vs. MSC^pH6.8^ CM)

Having demonstrated nerve recruitment and support by the OS microenvironment both in tissue samples and in in vitro models, we investigated the functional impact of this neural crosstalk on tumour aggressiveness. Neuron CM significantly enhanced OS cell (HOS and 143B) viability and proliferation, as shown by direct cell counting (Fig. 8A-B, *p* = 0.0003 and *p* < 0.0001, respectively) and Alamar blue assay (Fig. 8C, *p* = 0.0003 and *p* < 0.0001, respectively). Such pro-proliferative effect was replicated in direct neurons-OS co-cultures (Fig. 8D, *p* = 0.0087 for HOS, and *p* = 0.0260 for 143B). Furthermore, neurons stimulated OS cell migration in Transwell co-cultures (Fig. 8E-F, *p* = 0.0022 for both lines) and enhanced migratory distance/speed movements in direct co-cultures (Supplementary Fig. S12, *p* < 0.0001).

Given the increased neurotrophic potential of acidosis-exposed MSC (MSC^pH6.8^), we hypothesized that MSC^pH6.8^ may prime sensory neurons toward a pro-tumorigenic phenotype. Neurons exposed to MSC^pH6.8^ CM produced a secretome that significantly enhanced 143B proliferation compared to non-acidosis-exposed MSC (MSC^pH7.4^) CM (Fig. 5G, *p* = 0.0499). Crucially, IL-6 blockade by TCZ reversed this effect (*p* = 0.0070 vs. MSC^pH7.4^ CM; *p* = 0.0002 vs. MSC^pH6.8^ CM). 

**Fig. 9:**
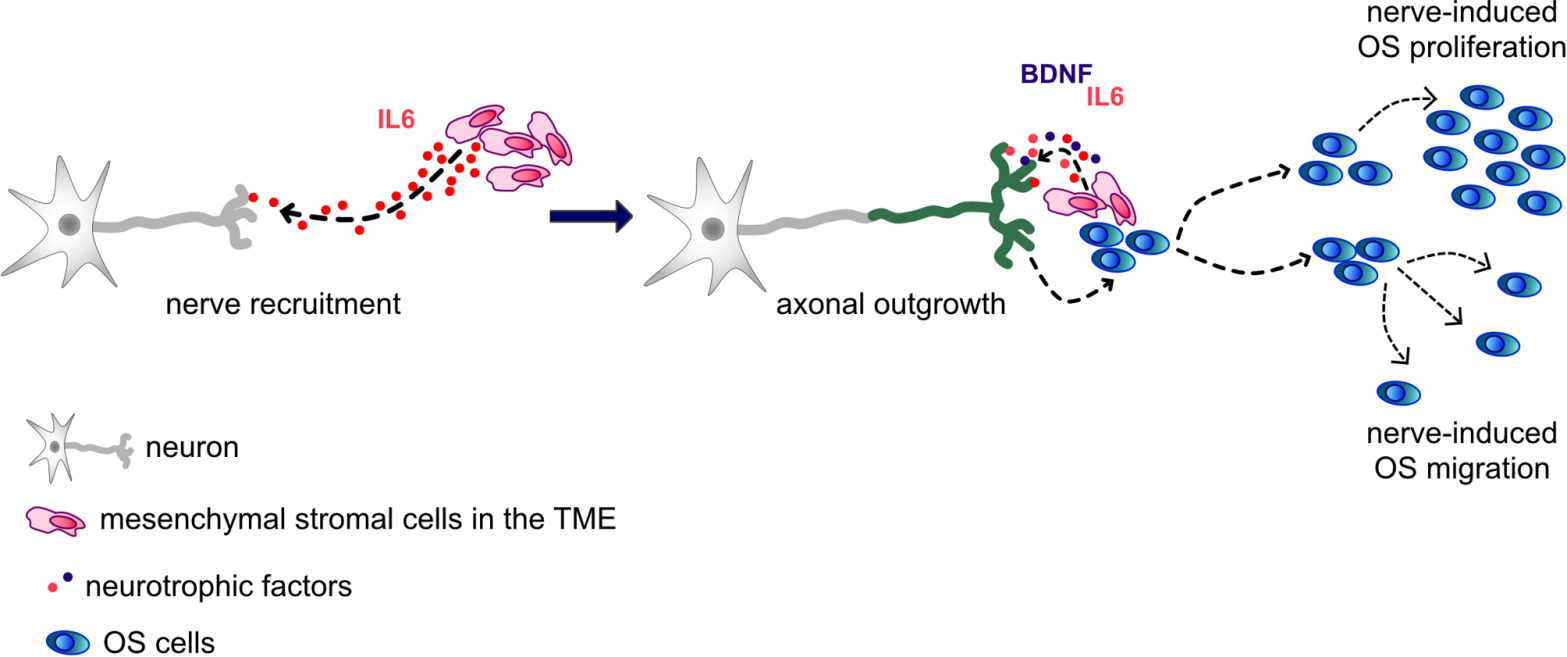
Proposed mechanism of nerve-stroma-OS crosstalk. Cancer-associated acidosis stimulates mesenchymal stroma to secrete pro-neurogenic mediators (e.g. IL-6), promoting nerve recruitment to the OS site. Tumour-associated mesenchymal cells and OS cells further enhance axonal outgrowth via neurokines (e.g. BDNF, IL-6) establishing a feedforward loop that drives OS proliferation and migration

In summary, neurons recruited to the OS microenvironment may drive tumour progression, particularly under acidosis. This crosstalk is mediated by IL-6 and potentially BDNF, stromal cells acting as central drivers (Fig. 9).

## Discussion

Nerve fibre outgrowth, a critical process in tissue repair and regeneration [45–47], exerts a key role in cancer biology, as suggested by suppressor effects of denervation on tumour growth and metastasis [48]. In OS, the pathogenetic function of innervation is largely unexplored, with research limited to analysing nerve-related molecule expression in in vitro models or tumour tissues [13–15]. This gap may exist because OS is a heterogeneous disease. It often includes regions with varying levels of differentiation, blood supply, oxygen, and glycolytic activity. A thorough assessment of tumour innervation requires sampling across multiple regions and specimens—an approach not feasible with biopsy material alone. In standard clinical practice, human OS is treated with neoadjuvant chemotherapy before surgical resection. This significantly alters tumour morphology and the TME, making it difficult to study native cellular interactions. To study nerve infiltration within the OS TME, we analysed tissue samples of canine OS. Investigating this naturally occurring disease, frequently affecting immunocompetent, large-size breeds, offers several advantages: (1) a striking biological and histological similarity to human OS [49, 50], (2) easy access to large series of patients, and 3) no interference with chemotherapy-induced artefacts.

Our study revealed βIII-tubulin-positive nerve fibres within OS tissues, consistent with previous observations in other solid tumours [9, 10, 51, 52]. We used βIII-tubulin-positivity as a reliable marker of peripheral nerves and axon outgrowth in other cancers [53–55]. The observed fibres appeared individual, disorganised, and with a branching pattern rather than established bundles infiltrating the tumour tissue. This pattern contrasts with the hypothesis of tumour cells infiltrating pre-existing nerves along the perineural space [56], instead suggesting active tumour-driven nerve recruitment and neo-axonogenesis [16]. Notably, βIII-tubulin-positive nerves showed strong and significant co-localisation with reactive stromal cells, hinting at a potential role of tumour-associated stroma in guiding neural infiltration. In the same tumour sections, the presence of neuronal axons also significantly correlated with an increased Ki-67 index in adjacent tumour cells, indicating a possible link between innervation and tumour cell proliferation.

To confirm the presence and localization of nerves in human OS, we analysed selected cases using the same methodology. From the long-term cohort at IRCCS Rizzoli Orthopaedic Institute, we identified five OS specimens from relapsed patients who underwent amputation without prior chemotherapy. Although rare, this clinical scenario offered a valuable chance to study the untreated TME. Immunohistochemical analysis for βIII-tubulin revealed innervation patterns consistent with those observed in the canine OS model. We found a marked increase in nerve fibres, especially in stroma-rich areas, including the peritumoral region near the periosteal reaction.

These findings support the clinical relevance of our results by confirming the presence of nerve fibres within the OS TME, particularly near mesenchymal stroma and proliferating cancer cells. However, establishing a statistical correlation between innervation and clinical outcomes in OS remains difficult. The tumour is highly aggressive in both humans and dogs. It shows high metastatic potential, rapid progression, and often presents with micrometastases at diagnosis, all of which complicate staging and prognosis [57].

While prior studies attribute nerve recruitment to cancer cell-derived neurotransmitters and neurotrophins [17], our study raises the possibility that mesenchymal stroma directly orchestrates this process. Stromal cells, known for their neurotrophic, pro-inflammatory activity, and consistent regenerative capacities [37, 38], perceive OS as an unhealed wound [3] and secrete factors that promote axonal growth. Conversely, neurons may reciprocally activate stromal cells, establishing a self-reinforcing loop [48]. This dynamic is further contextualised by the mesenchymal origin of OS, where stromal elements dominate the TME and likely retain intrinsic neurotrophic functions.

To elucidate the role of mesenchymal stroma in OS innervation, we focused on bone marrow MSC and OS-derived CAF. Bone marrow MSC exhibit unique neurotrophic properties compared to those from other sources [58], with a higher propensity to be recruited into the OS microenvironment. In our study, in co-culture models, MSC established more neuronal contacts than OS cells, both in standard cultures and in microfluidic axonal isolation systems. We use this type of support since microfluidic devices provide spatial and temporal control of the neuronal microenvironment, particularly for studying axonal elongation. Their design, featuring shallow microgrooves and hydrostatic pressure-driven fluidic isolation, restricts soma migration while guiding axons into dedicated compartments [59, 60]. Both MSC and CAF significantly promoted axonal attraction, branching, and elongation, often surpassing the effects of OS cells. Additionally, MSC demonstrated a bidirectional chemotactic response, as they were also attracted to neurons. This highlights their potential to drive OS axonogenesis and based on the existing literature. This phenomenon was also confirmed when we employed a second microfluidic platform to investigate interactions between neurons and tumour/stromal cells in a 3D context. This device incorporated heterotypic spheroids composed of MSC/143B cells and neurons. Compared to conventional 2D cultures, such systems more accurately replicate the in *vivo* TME by preserving the 3D architecture, nutrient gradients, and stromal interactions that influence tumour behaviour. Previously, we successfully used a similar platform containing OS cells and tumour-associated mesenchymal stromal cells to study tumour cell migration under physiologically relevant conditions [39].

Axonal attraction in the OS TME, likely driven by tumour-associated stroma, appears to be mediated by neurotrophic factors such as NGF and BDNF, key regulators of neuronal survival, differentiation, and outgrowth during nerve regeneration [61–63]. Notably, NGF and BDNF are upregulated across multiple cancers, including OS [64, 65], where they correlate with enhanced tumour innervation, disease progression, and poor outcome [66–70]. In this study, we focused on NGF, BDNF, and IL-6 as they are the most prominently expressed in the OS microenvironment, as previously demonstrated [65, 71, 72]: NGF and BDNF are consistently detected in OS tissues, with immunohistochemical analyses revealing strong positivity in most patient samples, and correlation with clinical outcome. Other neuromodulators—such as glial cell line-derived neurotrophic factor (GDNF), neuropilin-2 (NRP-2), or neurotrophins (NT3 and 4)—have also been investigated [64, 73, 74]. However, these findings are less frequent and mostly limited to preclinical models, with little translational confirmation [75]. We also confirmed the neuromodulatory activity of IL-6 and BDNF in our experimental setting. Both factors caused a dose-dependent increase in axonal growth, with IL-6 showing the strongest effect. Furthermore, in our study, MSC and CAF secreted significantly higher levels of BDNF compared to OS cells. In contrast, although high levels of NGF have been previously reported in OS tissue [13, 64, 65], we detected NGF exclusively in the MSC secretome. These findings suggest that BDNF acts as the principal stromal-derived nerve chemoattractant in OS, with NGF playing a minor role. Notably, both MSC and CAF secreted high levels of IL-6. Although not classically defined as a neurotrophic factor, IL-6 enhances neuronal survival and outgrowth in DRG neurons [76, 77] and other neuronal subtypes [78] via modulation of neurotrophic signalling [78, 79]. Beyond its established role in driving OS proliferation and migration [40, 80], in this study we also demonstrated that MSC-derived IL-6 promotes sensory nerves growth, branching, and axonogenesis, while IL-6 blockade significantly decreased axonal outgrowth. However, a similar effect was also found after the impairment of BDNF stimulation.

Extracellular acidosis, a hallmark of aggressive tumours driven by a dysregulated metabolism [81], worsens OS progression by enhancing IL-6 secretion by MSC via the NF-κB inflammatory pathway [3, 39]. Acidosis also activates pain-sensitive nerves in bone malignancies via acid-sensing ion channels [82, 83]. Here, we showed that acidic conditions further potentiate MSC-mediated axonal growth, likely via IL-6 and BDNF, which is similarly upregulated under acidosis [62]. Notably, the tumour cell secretome—contained in conditioned medium from OS cells—elicited a comparable effect, even in the absence of pH alterations, suggesting that tumour-derived factors can independently promote axonal outgrowth.

Finally, using co-cultures and CM models – established tools to investigate paracrine nerve-tumour interactions [84, 85] – we demonstrated that both neuron-derived secretome and direct neuronal contact enhance OS proliferation and migration, mirroring previous observations in carcinomas [10, 86, 87]. Critically, neurons primed by acid-stressed MSC secretome amplified tumour viability in an IL-6-dependent manner, underscoring the central role of IL-6 in the axonogenesis induced by MSC of OS stroma.

Given the complexity and dynamic nature of the TME, we investigated whether other cell types—specifically monocytes and osteoblasts—also contribute to the secretion of neurotrophic factors such as BDNF and IL-6 [88, 89]. Conditioned media from both cell types promoted axon elongation in sensory neurons, an effect that was reversed by blocking IL-6 and BDNF. These results indicate that multiple cell types can support axonogenesis within the OS TME, likely via distinct mechanisms. While osteoblasts are involved in normal bone physiology, monocytes and macrophages are more closely linked to inflammation and tissue repair. In the OS TME—characterized by chronic inflammation and accelerated bone remodelling—MSC, osteoblasts, and monocytes/macrophages may act together to establish a pro-innervating environment.

Although our findings confirm the involvement of various cell types, histological analysis suggests that MSC are the most relevant and direct source of chemotactic signals guiding nerve growth in our model. However, our results do not exclude the contribution of other cells. Rather, they emphasize a specific and functionally validated interaction between MSC and sensory nerves in the context of tumour innervation.

## Conclusions

Our study uncovers novel insights into nerve infiltration in OS, revealing a dynamic interplay between neurons, mesenchymal stroma, and tumour cells that shapes the TME. We provide the first evidence that stromal cells in the TME orchestrate nerve recruitment and outgrowth toward OS via the secretion of neurotrophic and pro-inflammatory factors, notably BDNF and IL-6. This finding lays the groundwork for exploring additional cellular components of the OS TME - such as immune and endothelial cells [18]–which may play critical roles in regulating tumour innervation and progression, as observed in other cancers. It also highlights the potential for novel therapeutic strategies in OS, including targeting IL-6 and BDNF, or developing more personalised approaches targeted at specific cell populations. These strategies may help disrupt stroma-driven axonogenesis and represent a promising avenue for slowing OS progression.

## Supplementary Information

Below is the link to the electronic supplementary material.

Supplementary Material 1

Supplementary Material 2

## Data Availability

All data generated or analysed during this study are included in this article and its supplementary information file.

## Abbreviations

OS: Osteosarcoma
TME: Tumour microenvironment
NGF: Nerve growth factor
BDNF: Brain-derived neurotrophic factor
MSC: Mesenchymal stromal cells
Alpha-MEM: Minimum Essential Medium Eagle Alpha Modified
CAF: Cancer-associated fibroblasts
IMDM: Iscove’s Modified Dulbecco’s Medium
FBS: Fetal bovine serum
DRG: Dorsal root ganglion
NSF-1: Neural cell-survival factor
cNBM: Complete Neuron Basal medium
CM: Conditioned medium
PDMS: Polydimethylsiloxane
IL-6: Interleukin-6
Anti-BDNF Ab: Anti-BDNF receptor antibody
TCZ: Tocilizumab
BCA: Bicinchoninic acid
SEM: Standard error of the mean
ACTA2: Smooth muscle alpha (α)-2 actin
FAP: Fibroblast Activation Protein Alpha
ECM: Extracellular matrix
MSC^143B CM^: Tumour-activated MSC
MSC^pH6.8^: Acidosis-exposed MSC
MSC^pH7.4^: Non-acidosis-exposed MSC
GDNF: Glial cell line-derived neurotrophic factor
NRP-2: Neuropilin-2
NT3: Neurotrophin-3
NT4: Neurotrophin-4
