# Supplementary material for: Mesenchymal stroma drives axonogenesis and nerve-induced aggressiveness in osteosarcoma
Source: J Exp Clin Cancer Res. 2025 Sep 30;44:276. doi: 10.1186/s13046-025-03532-2 (PMC12487423; doi:10.1186/s13046-025-03532-2)
Supplement: Supplementary file 1 — Supplementary Material 1 [file 13046_2025_3532_MOESM1_ESM.pdf]

Summary

SUPPLEMENTARY MATERIALS AND METHODS 2

Immunohistochemistry for βIII-tubulin and Ki-67 and relative quantification 2

***β*III-tubulin immunofluorescence *s*taining.** 4

Standard co-cultures of sensory neurons and MSC/143B. 4

Static cultures of sensory neurons 4

MSC activation by OS cell culture supernatant 4

Molecular characterization of CAF 5

Migration assay in neurons-OS direct co-culture 5

3D Cell Cultures: Microfluidic Models 6

Migration of MSC towards neurons: time-lapse imaging and transwell assay. 6

Time-lapse assay. 6

Transwell migration assay. 7

Axonal growth quantification in response to monocyte- and osteoblast-conditioned media. 7

Human $CD14^+$ monocytes cultures. 7

Human osteoblasts (hOB) cultures. 8

Preparation of CM. 8

Axonal growth quantification. 8

SUPPLEMENTARY FIGURES 9

Supplementary Fig. S1. Schematic diagram of the protocol for obtaining conditioned media and testing their effects on neuronal growth. 9

Supplementary Fig. S2. Experimental setup for axonal recruitment. 10

Supplementary Fig. S3. βIII-tubulin staining of nerve fibres in canine OS tissues. 11

Supplementary Fig. S4. Spatial distribution of βIII-tubulin-positive nerves in canine OS tissues. 12

Supplementary Fig. S5. Comparison of innervation in human OS tissues and healthy human bones. 13

Supplementary Fig. S6. Negative and positive controls for βIII-tubulin immunohistochemistry. 15

Supplementary Fig. S7. Immunofluorescence of neurons-OS cells co-culture. 16

Supplementary Fig. S8. CAF molecular characterization. 17

Supplementary Fig. S9. 3D Microfluidic model of the innervated tumour microenvironment. 18

Supplementary Fig. S10. Effect of DRG Neurons on MSC Migration. 19

Supplementary Fig. S11. Axonal outgrowth promoted by osteoblasts and monocytes is mediated by IL-6 and BDNF. 20

Supplementary Fig. S12. Effect of DRG Neurons on 143B OS Cell Migration. 21

Supplementary Materials and Methods

Immunohistochemistry for βIII-tubulin and Ki-67 and relative quantification

Paraffin-embedded sections derived from canine and human OS and normal bone tissues were incubated o.n. with the rabbit polyclonal anti-beta III Tubulin primary antibody (ab18207, Abcam, Cambridge, UK, 1:1600 and 1:4000 for canine and human tissue sections, respectively), followed by washing and incubation with a biotinylated secondary antibody. Staining was developed using DAB and sections counterstained with Mayer's haematoxylin (EnVision FLEX, High pH Link visualization system, Agilent Technologies, Santa Clara, CA, USA). Negative controls included sections treated with the diluent buffer in place of the primary antibody and tissue-based negative control using canine liver. Human peripheral nerve tissue was used as a positive control (Person F, et al Tumour Biol. 201;39:1010428317712166). For Ki-67 staining, canine OS sections were incubated o.n. with a mouse monoclonal anti-Ki-67 primary antibody (1:1500, M7240, Agilent Technologies, Santa Clara, CA, USA), followed by the same detection and counterstaining protocol as above.

Digital images of all stained sections were acquired at 20× magnification using an Eclipse E800M microscope (Nikon). Quantitative analysis was performed using ImageJ software. After colour deconvolution to isolate the DAB signal, a consistent threshold was applied across all images. For Ki-67 analysis, Ki-67-positive cells were manually counted in 10 randomly selected fields. The Ki-67 index was calculated as the percentage of Ki-67-positive cells relative to the total number of tumour cells.

To quantify nerve fibre density based on βIII-tubulin staining, βIII-tubulin-positive nerve fibres were manually counted in at least five innervated regions per sample. Nerve fibres were defined as distinct linear or circular structures consistent with axonal morphology. Data were normalised by tissue area and expressed as nerve fibres per square millimetre (n/mm^2, innervation index), providing a robust metric for comparison across samples. Based on the distribution of innervation

indices in our canine cohort, we defined four categories of innervation using percentile thresholds (Table 2). The category, based on the percentile distribution of the innervation index, were set as follows: 0 for 'Absent' (None); > 0 to ≤ 4.923 (25th percentile) for 'Weak' (+); > 4.923 (25th percentile) to ≤ 35.12 (75th percentile) for 'Moderate' (++); > 35.12 (75th percentile) for 'Strong' (+++) innervation.

To further explore the presence of tumour innervation within the stroma and tumour aggressiveness, we examined the spatial distribution of nerve fibres in relation to stromal and tumoural regions. Due to the overlapping antigenic profiles between mesenchymal stromal cells and tumour cells in OS, it is not possible to distinguish them reliably on a cell-by-cell basis using specific markers. Therefore, stromal regions were identified based on morphological criteria—namely, the presence of fibroblast-like cells with elongated, uniformly stained (non-pyknotic) nuclei forming characteristic stromal structures such as dense fibrous septa or membranous patterns. These features are consistent with desmoplastic reactions, which reflect the activity of mesenchymal stromal cells (differentiated into myofibroblasts) attempting to repair tissue and restrict tumour expansion (Ariza-Prota MA et al., Respir Med Case Rep 2015;16:112–6; Chirumbolo S et al., Int J Clin Oncol 2017;22:222–8; Ueno H et al., Am J Surg Pathol 2017;41:1506–12). To assess the spatial distribution of nerves relative to the tumour stroma and anatomical regions, we quantified βIII-tubulin-positive axons located within the tumour core versus the periphery using ImageJ. We also classified nerve fibres based on their proximity to mesenchymal stroma: stromal-associated (βIII-tubulin-positive fibres located within a 25 μm radius of a mesenchymal-stromal-like cell nucleus), and stromal-distal (fibres located beyond this radius or in tumour regions lacking mesenchymal stroma). Mesenchymal stromal-like cells were identified morphologically under brightfield microscopy as fibroblast-like cells with elongated, uniformly stained nuclei.

β-III tubulin immunofluorescence staining

Standard co-cultures of sensory neurons and MSC/143B

Co-cultures were fixed with 3.7% paraformaldehyde, permeabilized with 0.1% Triton X-100, and blocked with 1% BSA. Primary rabbit anti-βIII tubulin (1:1000, ab18207, Abcam, Cambridge, UK) and mouse monoclonal anti-vimentin (1:100, sc6260, Santa Cruz Biotechnology, Dallas, Texas, USA) antibodies were incubated o.n. at 4°C. Alexa Fluor 568 anti-rabbit and Alexa Fluor 488 anti-mouse (Life Technologies) secondary antibodies were used, plus Hoechst 33258. Images were acquired by Nikon Eclipse E800 fluorescence microscope (Nikon, Japan, lens 40x).

Static cultures of sensory neurons

In a subset of experiments with static neuron monocultures, neurons were immunostained for βIII-tubulin by immunofluorescence to visualize axons (see "Axonal growth quantification" in the manuscript). Briefly, after the 72 h incubation with CM, neurons were washed and fixed with 3.7% paraformaldehyde, permeabilized with 0.1% Triton X-100, blocked with 1% BSA, and immunostained with rabbit polyclonal anti-βIII tubulin primary antibody (1:1000, ab18207, Abcam, Cambridge, UK) and with Alexa Fluor 488 secondary antibody (Life Technologies), plus Hoechst 33258 for nuclei counterstaining. Axonal growth was quantified from images obtained with a fluorescence microscope (10x lens) and using the ImageJ software.

MSC activation by OS cell culture supernatant

Conditioned medium (CM) from 143B cells (143B CM) was used to activate MSC and obtain their CM for the evaluation of axonal growth in static cultures. Briefly, 143B CM was obtained after 72 h incubation of 143B cells (70% confluency) with Alpha-MEM plus 0.1% FBS (low-serum medium), subsequent collection, and centrifugation at 6000 rpm for 15 min at RT.

MSC were seeded in T25 cell culture flasks (3×10^5 cells/flask) in standard conditions. After adhesion, cells were washed with PBS and cultured for 10 h in low-serum medium. Then, cells were washed again with PBS and incubated for 24 h with fresh 143B CM to obtain $MSC^{143B\ CM}$

CM. At the end of incubation, cells were washed again with PBS and cultured for additional 48 h in complete Neuron Basal medium without NSF-1. The supernatant was then collected, centrifuged to remove cellular debris, concentrated using Vivaspin 20 sample concentrator (10 kDa MWCO; GE Healthcare, Chicago, IL, USA) by centrifugation at 6000 rpm for 15 min at +4°C, and stored at −80°C until use.

Molecular characterization of CAF

To validate the CAF phenotype, we analysed mRNA expression of ACTA2 and FAP (Nurmik M et al., Int J Cancer. 2020;146(4):895-905) by quantitative reverse transcription polymerase chain reaction (qRT-PCR). Total RNA was isolated from CAF and normal human skin fibroblasts using TRIzol reagent (Invitrogen, Thermo Fisher Scientific) and reverse transcribed with MuLV reverse transcriptase (Applied Biosystems, Thermo Fisher Scientific). qRT-PCR was performed by amplifying 1 μg of cDNA using the light cycler instrument and the Universal Probe Library system (Roche Applied Science). Primer sequences and probes were: Hydroxymethylbilane synthase (HMBS, accession number NM_000190.3), F = tgtggtgggaaccagctc, R = tgttgaggtttccccgaat, probe 26; ACTA2 (accession number NM_001141945.1, NM_001613.2), F = tcatgatgctgttgtaggtggt, R = ctgttccagccatccttcat, probe 58, FAP (accession number NM_004460.2), F = atccgaacaacgggattctt, R = tggcgatgaacaatatcctaga, probe 19.

Migration assay in neurons-OS direct co-culture

143B and HOS cells were seeded (1.8×10^3 and 2×10^3 cells/well, respectively) in pre-coated 48-well plates in complete IMDM. After 7 h of culture, medium was discarded, and neurons (1.8×10^3 cells/well) were added in complete neuronal basal medium (cNBM). After 24 h of co-culture, time-lapse imaging was performed using ImageXpress Pico. The total distance covered by OS cells and the mean migration speed were automatically quantified and compared to monocultures using the ImageJ software. This approach involves analysing time-lapse microscopy images, allowing for

precise tracking of cellular movement and accurate calculation of both parameters. For each technical replicate, measurements were performed on at least 100 different OS cells in both monocultures and co-cultures.

3D cell cultures: microfluidic models

We obtained heterotypic spheroids (143B OS cells/MSC) by seeding cells in ultralow attachment plates (SBio, NH, USA) in a 1:3 ratio (5×10^3 tumour cells + 1.5×10^4 stromal cells) in 200 µL RPMI 1640 medium/well; spheroids were then grown for 96 h under standard conditions (37°C, 5% CO_2, humidified atmosphere). At this time-point, the spheroids were placed in the open grafting chamber of a microfluidic OrganoPlate® Graft (Mimetas, Oegstgeest, The Netherlands), previously filled with 75% Matrigel® (BD Life Sciences, Biosciences, Franklin Lakes, NJ, USA) and 25% cell culture medium supplemented with 0.1% FBS. The chamber was then covered by 0.1% FBS medium. DRG neurons were seeded (4×10^3 cells) in cNBM in the two lateral channels. The stromal-tumour spheroid and neurons were then co-cultured for 7 days. At the endpoint, cells were fixed with 3.7% paraformaldehyde, βIII-tubulin immunofluorescence staining was performed as described in the Supplementary Information, and nuclei were stained with Hoechst 33258 (Sigma). Images were acquired with an objective 20x air, numerical aperture 0.75, Resonant scanning, zoom at 1, line average of 4 (A1R MP confocal microscope, Nikon, scale bar 50 µM).

Migration of MSC towards neurons: time-lapse imaging and transwell assay

Time-lapse assay

MSC were seeded (1.8×10^3 cells/well) into pre-coated 48-well plates in complete Alpha-MEM. After 24 hrs from adhesion, the medium was replaced with cNBM containing neurons (1.8×10^3 cells/well). After additional 24 hrs from neuron adhesion, we conducted a 6-hour time-lapse live-cell imaging experiment using the ImageXpress Pico system to directly visualise the dynamics of MSC–neuron co-culture. For the analysis of the assay, neurons were readily distinguishable by their

smaller size and characteristic pseudounipolar morphology, featuring a single axon bifurcating into two extensions. For clarity, MSC were marked with blue dots and neurons with red dots in the time-lapse video.

Transwell migration assay

Neurons (1.5×10^4 cells) were seeded in pre-coated 24-well plates and cultured in cNBM. Wells containing cNBM alone served as negative controls. After 48 hours, MSC (1.5×10^4 cells) were seeded into 8 µm-pore Transwell inserts and placed into wells containing either neuron-free medium (−neurons) or pre-cultured neurons (+neurons). After an additional 48 hours, migrated MSC were fixed with methanol, stained with crystal violet, and counted in nine random fields per insert (20× objective). The assay was performed in triplicate across two independent experiments.

Axonal growth quantification in response to monocyte- and osteoblast-conditioned media

Human $CD14^+$ monocytes cultures

$CD14^+$ cells were derived from peripheral blood mononuclear cells (PBMCs) isolated from buffy-coats of one healthy donor by immunomagnetic separation using an anti-CD14 monoclonal antibody (MiniMACS; Myltenyi Biotec, Bologna, Italy). Briefly, PBMCs were isolated from buffy-coats on Ficoll-Hystopaque gradient (GE Healthcare, Chicago, IL, USA), washed with MACS buffer (PBS at pH 7.2, supplemented with 0.5% bovine serum albumin and 2 mmol/L ethylene diamine tetraacetic acid), and clumps were removed by passing cells through a 30-µm prefilter. Cells were then centrifuged at 400 × g for 15 min. The cell pellet was suspended in MACS buffer (10^7 cells in 80 µl), mixed with 20 µl of anti-CD14 MACS antibody-coated microbeads (Miltenyi Biotec), and incubated for 15 min at R°T. The cell suspension was applied to an LS-positive selection column that was previously washed with 1 ml of MACS buffer and placed in a magnetic separation unit. The column was rinsed with 3.5 ml of MACS buffer, then removed from the magnetic separation unit, and positive bound cells were flushed with 2.5 ml of buffer. Human $CD14^+$ monocytes were maintained in high-glucose Dulbecco's modified Eagle's medium (DMEM)

supplemented with 10% heat-inactivated characterized FBS (Euroclone, Milan, Italy), plus 100 U/ml of penicillin, and 0.1 mg/ml of streptomycin (Life Technologies, Carlsbad, CA, USA) and cultured at 37°C, in a humidified atmosphere of 5% CO_2.

Human osteoblasts (hOB) cultures

hOB from healthy donors were purchased from VWR International PBI (Milan, Italy). hOB were maintained in DMEM supplemented with 10% FBS (Euroclone, Milan, Italy), plus 100 U/ml of penicillin, and 0.1 mg/ml of streptomycin (Life Technologies, Carlsbad, CA, USA) and cultured at 37°C, in a humidified atmosphere of 5% CO_2.

Preparation of CM

$CD14^+$ monocytes and hOB CM were obtained after 48 h incubation of cells (70% confluency) with NBM w/o NSF-1. The supernatant was then collected, centrifuged to remove cellular debris, concentrated using Vivaspin 20 sample concentrator (10 kDa MWCO; GE Healthcare, Chicago, IL, USA) by centrifugation at 6000 rpm for 15 min at +4°C, and stored at −80°C until use.

Axonal growth quantification

Neurons (7.5×10^3 cells/well) were seeded onto pre-coated chamber slides. After adhesion, cells were washed and incubated for 72 h with CM from $CD14^+$ monocytes or hOB. For interleukin-6 (IL-6) blocking experiments, neurons were pre-treated with 100 μg/ml the anti-IL6 receptor antibody (tocilizumab, TCZ) for 48 h prior to CM exposure, with treatments replenished every 24 h. For BDNF blocking experiments, neurons were pre-treated with 1 μg/ml anti-BDNF receptor antibody (Anti-BDNF Ab) (PeproTech, Cranbury, NJ, USA) for 48 h prior to CM exposure, with treatments replenished every 24 h. Axonal growth was quantified from brightfield images obtained with an optical microscope (10x lens) and using the ImageJ software.

Supplementary Figures

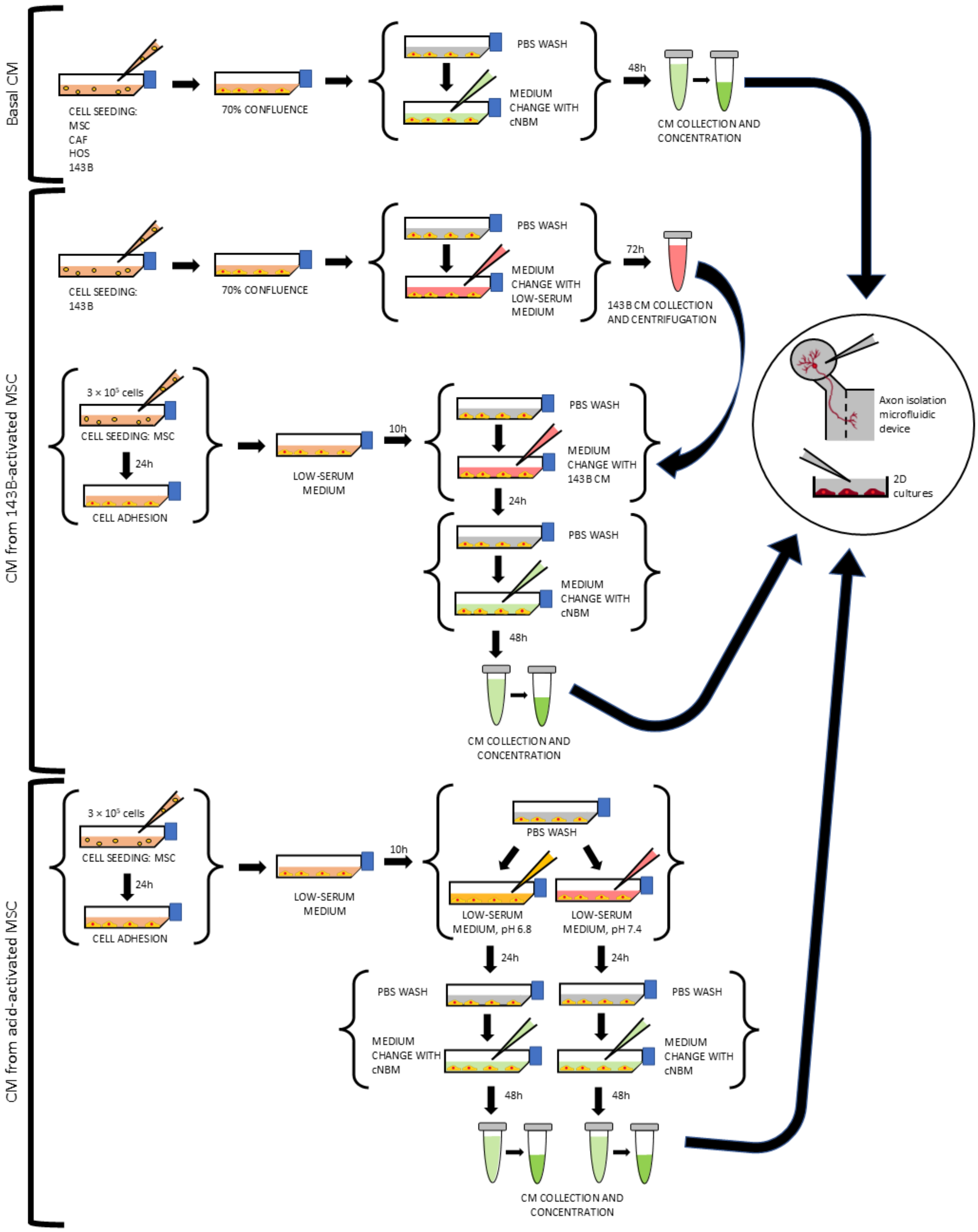

Supplementary Fig. S1. Schematic diagram of the protocol for obtaining conditioned media and testing their effects on neuronal growth.

Workflow to obtain the conditioned media, starting from standard and pre-treated cell cultures, and their application to neurons to evaluate axonal growth and recruitment in 2D cell cultures and microfluidic axon isolation devices.

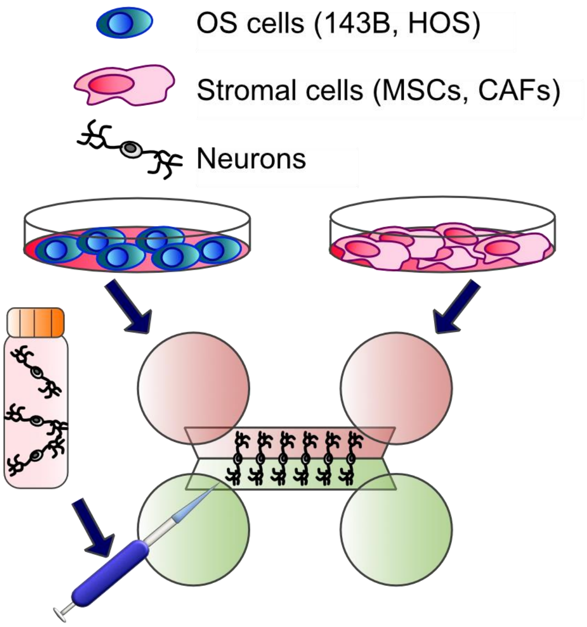

Supplementary Fig. S2. Experimental setup for axonal recruitment.

Neurons were seeded in the somal compartment (green), CM was added to the axonal compartment (red, stimulus).

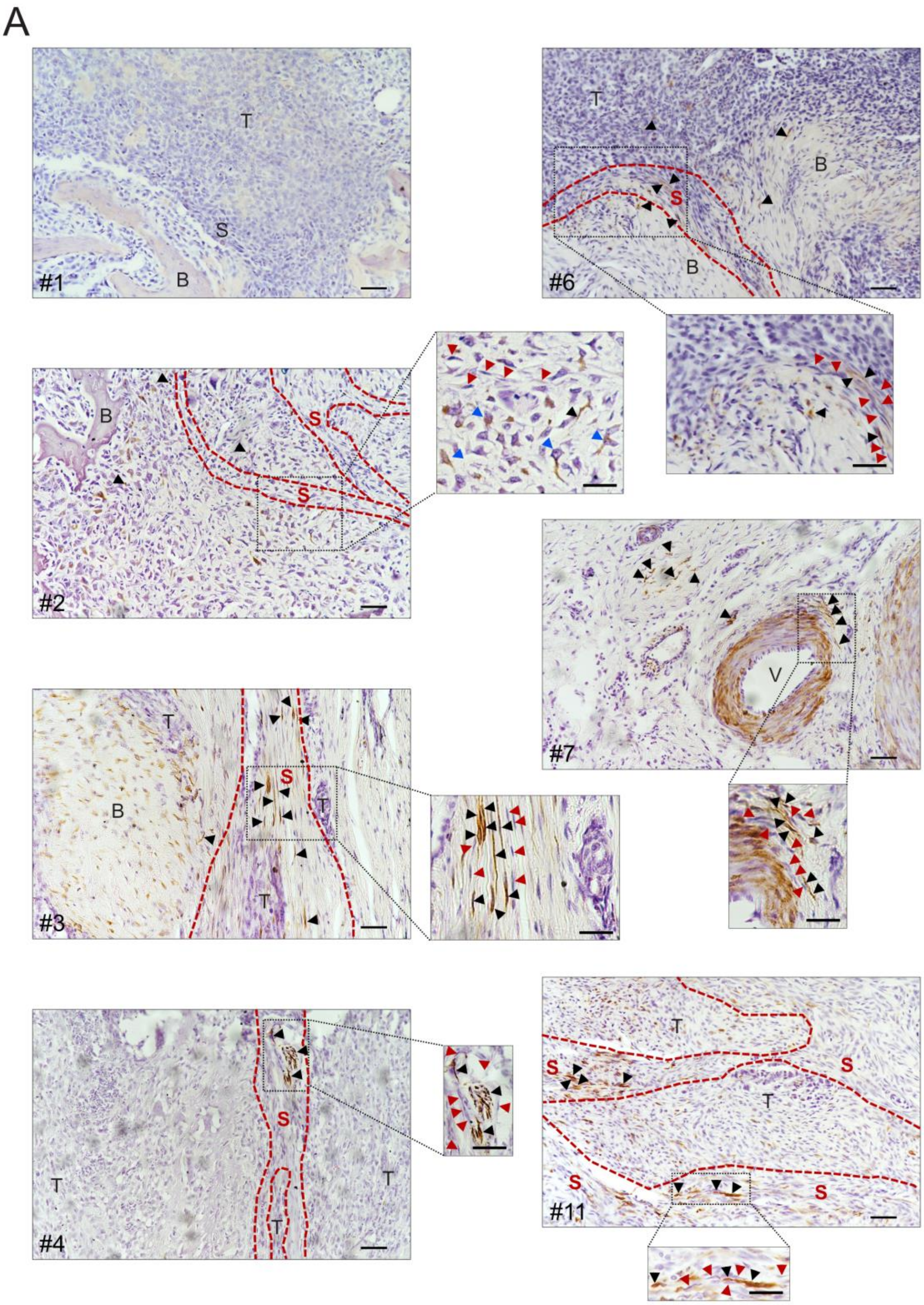

Supplementary Fig. S3. βIII-tubulin staining of nerve fibres in canine OS tissues.

(A) Representative images showing βIII-tubulin+ axons. High-magnification insets highlight fibres (black arrows) within stromal regions (S, stroma) adjacent to tumour-associated stromal cells (red arrows). Some fibres infiltrate the tumour parenchyma and appear at the bone-tumour interface (B, bone; T, tumour), as seen in samples #6 and #2. In sample #2, βIII-tubulin signal is also present in the cytoplasm of stromal and tumour cells (blue arrows). In sample #7, fibres surround vessels (V). Sample #1 shows no staining. Scale bar = 50 µm.

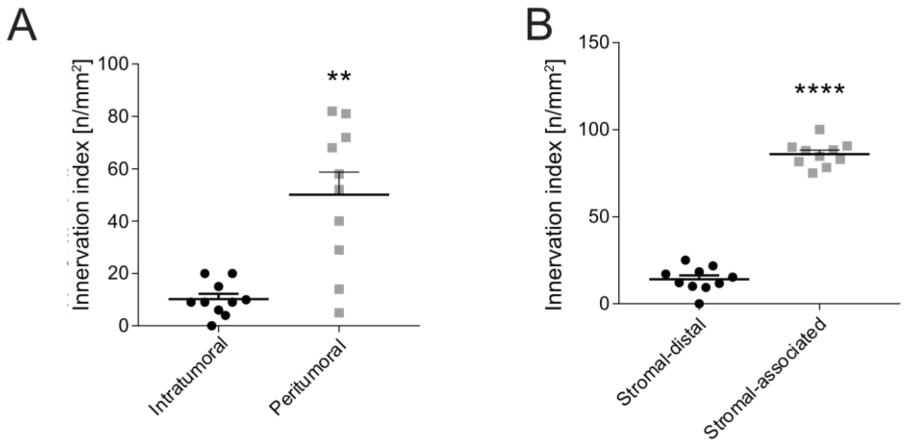

Supplementary Fig. S4. Spatial distribution of βIII-tubulin-positive nerves in canine OS tissues. **(A)** Number of βIII-tubulin-positive axons within the tumour (intratumoral) and at its periphery (peritumoral) (**$p < 0.01$). **(B)** Localization of βIII-tubulin-positive axons relative to the tumour stroma (distal vs associated) (****$p < 0.0001$). Data were expressed as nerve fibres per square millimetre (n/mm², innervation index), mean ± SE, N = 10 for both panels.

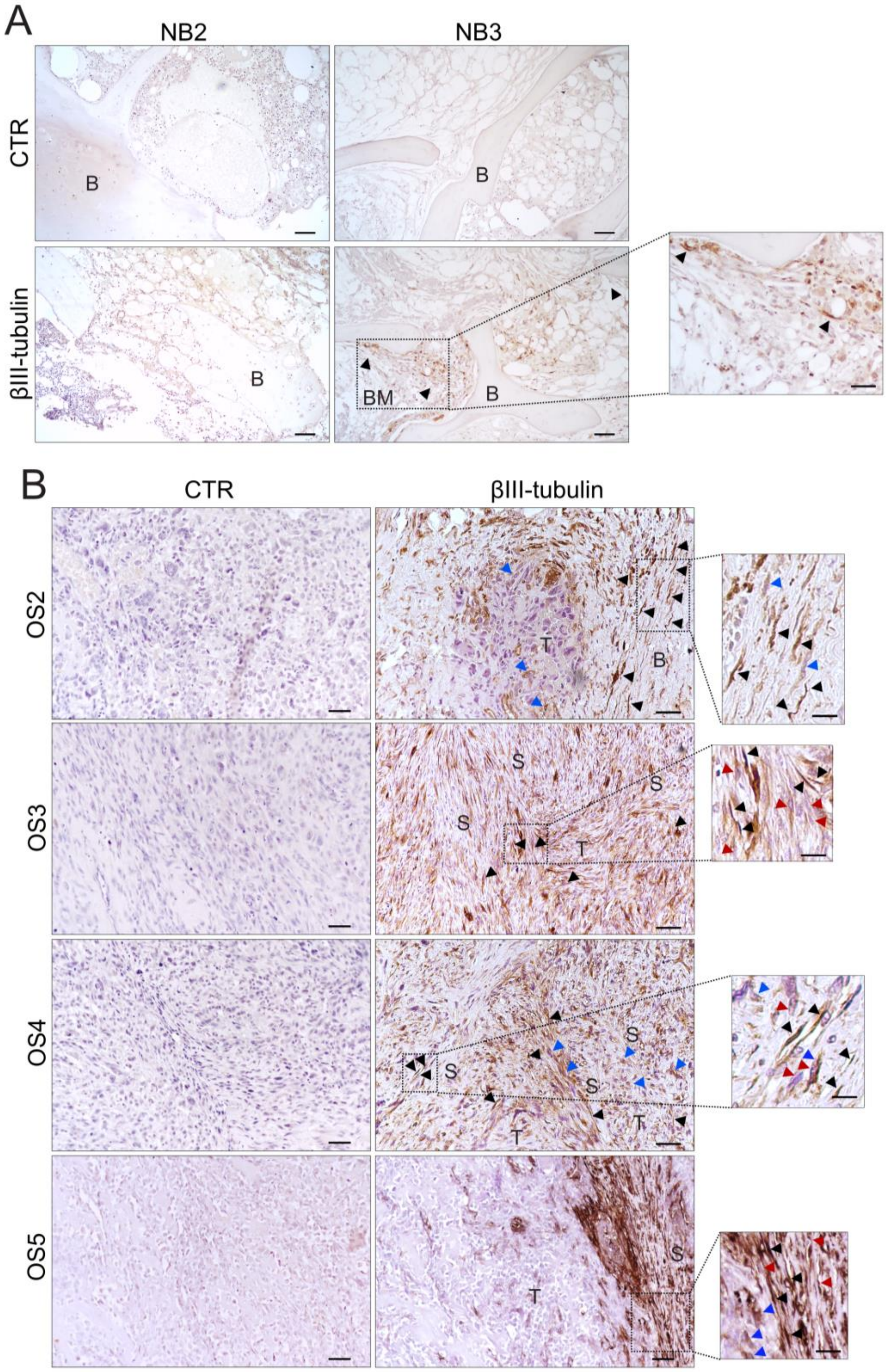

Supplementary Fig. S5. Comparison of innervation in human OS tissues and healthy human bones. Scale bar = 50 μm (B, Bone; BM, bone marrow; S, stroma; black arrows, βIII-tubulin+ nerve axons; red arrows, tumour-associated stromal cells). **(A)** Representative βIII-tubulin immunohistochemistry and corresponding negative control (CTR; sections incubated with diluent buffer w/o primary antibody) from two of three healthy bone samples. High-magnification insets show few βIII-tubulin+ axons at the bone-bone marrow interface in NB3 sample. **(B)**

Representative βIII-tubulin immunohistochemistry and corresponding CTR from four of five human OS samples. High-magnification insets highlight βIII-tubulin+ nerve axons within stromal regions and adjacent to tumour-associated stroma across three OS cases. In OS2, OS4, and OS5 samples, βIII-tubulin signal is also present in the cytoplasm of stromal and tumour cells (blue arrows).

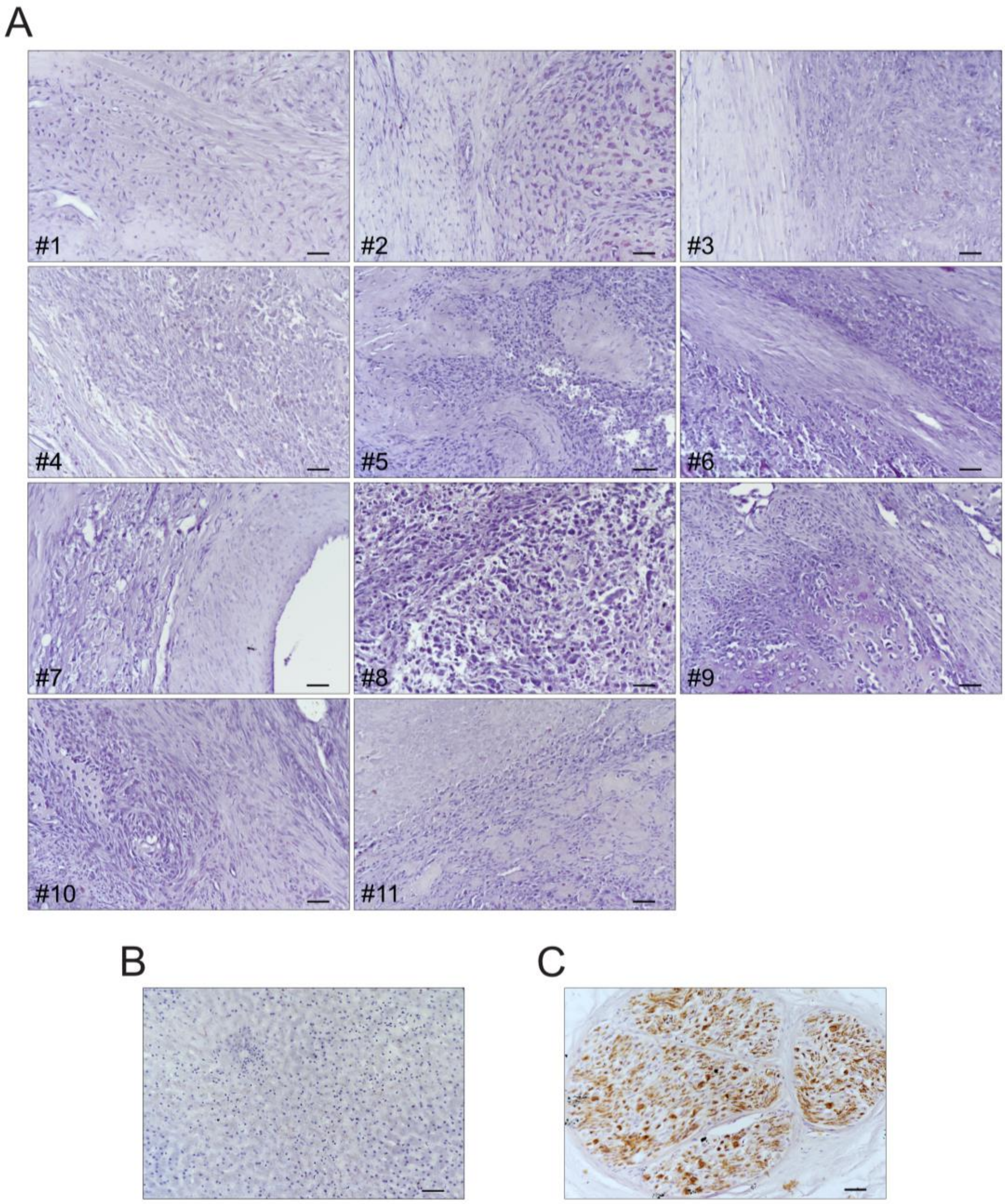

Supplementary Fig. S6. Negative and positive controls for βIII-tubulin immunohistochemistry.
(A) Representative images of negative controls: paraffin-embedded canine OS tissue sections incubated with diluent buffer w/o primary antibody. Canine liver **(B)** and human nerves **(C)** tissues were used as negative and positive controls, respectively, to validate antibody specificity. Scale bar = 50 µm.

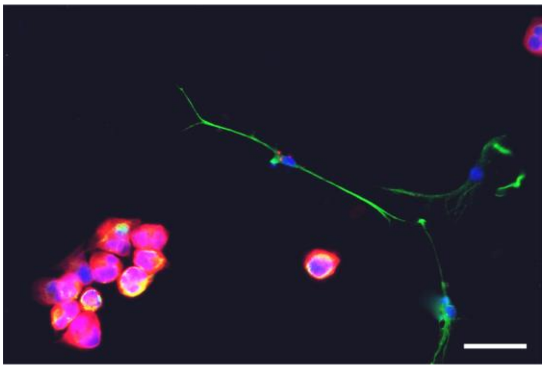

Supplementary Fig. S7. Immunofluorescence of neurons-OS cells co-culture.

β-III-tubulin+ neurites (green) extend toward vimentin+ 143B (red) (40×, scale bar 50 μm).

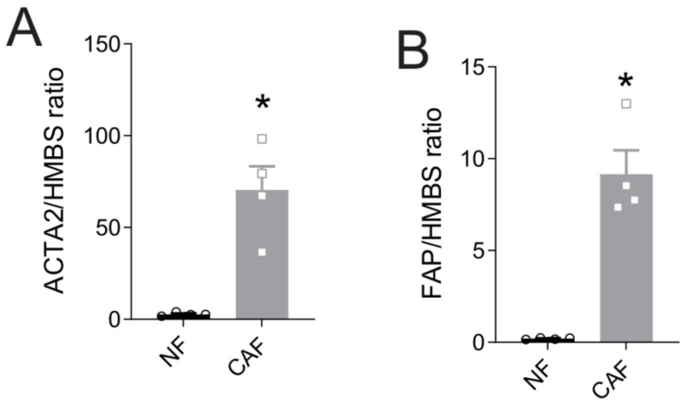

Supplementary Fig. S8. CAF molecular characterization.
(A) Q-RT-PCR analysis of smooth muscle alpha (α)-2 actin (ACTA2) and **(B)** Fibroblast Activation Protein Alpha (FAP) revealed that CAF isolated from an OS tissue have significantly higher expression of these typical markers than normal human dermal fibroblasts (NF) (mean ± SE, 2 inter-assay and 2 intra-assay replicates, N = 4, * $p < 0.05$).

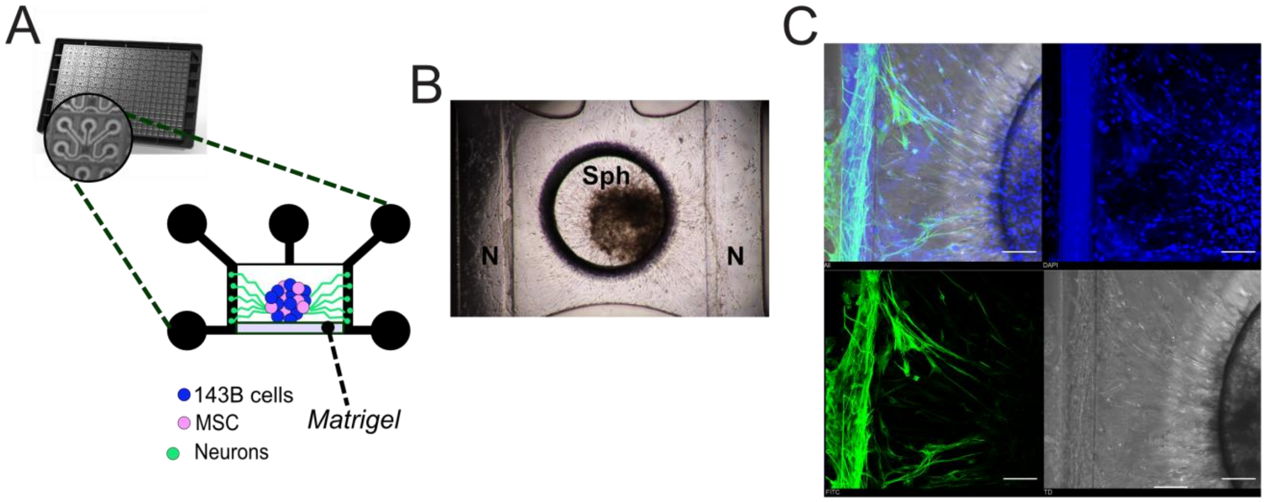

Supplementary Fig. S9. 3D Microfluidic model of the innervated tumour microenvironment. **(A)** Schematic representation of the experiment conducted using the OrganoPlate Graft® system. A heterotypic tumour spheroid composed of MSC and 143B cells was embedded in extracellular matrix (ECM) and positioned in the central chamber of the chip. Neurons were seeded in the adjacent lateral channels and allowed to extend axons towards the ECM and the spheroid. **(B)** Representative image from the inverted optical microscope showing the spheroid (Sph) located in the central chamber and neurons (N) in the lateral channels. **(C)** Representative confocal images of the spheroid after 7 days of co-culture, stained with Hoechst 33258 (blue) and βIII-tubulin immunofluorescence (green), highlighting numerous axonal projections extending from the neurons in the lateral channels towards the spheroid. Images were acquired using a 20× air objective (NA 0.75), resonant scanning, zoom 1, line average 4, on a Nikon A1R MP confocal microscope. Scale bar: 50 µm.

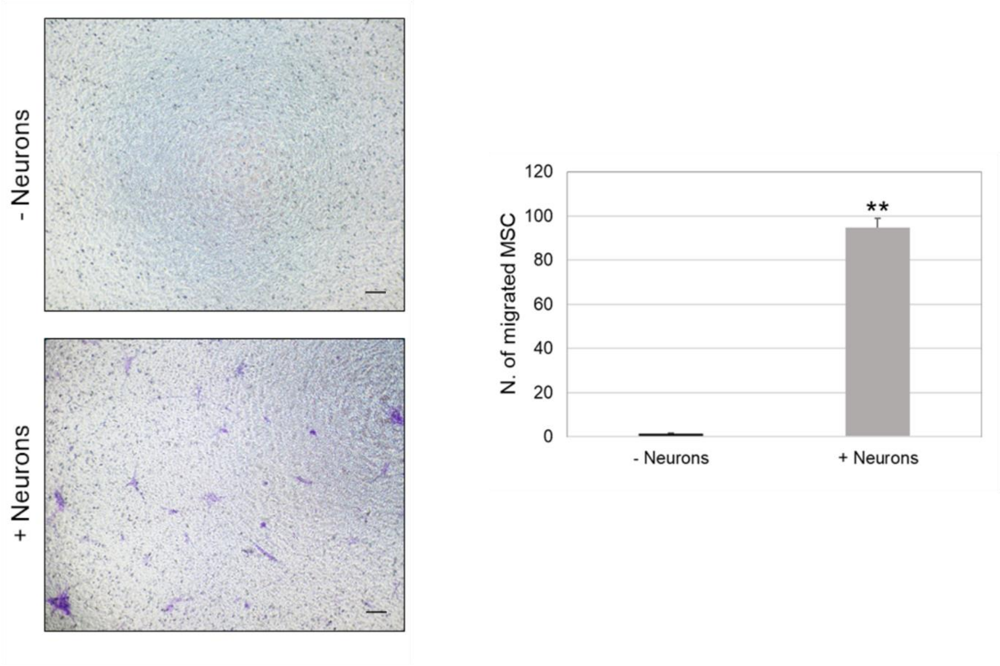

Supplementary Fig. S10. Effect of DRG Neurons on MSC Migration.
Left, representative images of crystal violet-stained migrated MSC on transwell membrane (20x, scale bar 100 μm). Right, quantification of migrated cells (means ± SE, 2 inter-assay and 3 intra-assay replicates, N = 6, **$p < 0.01$).

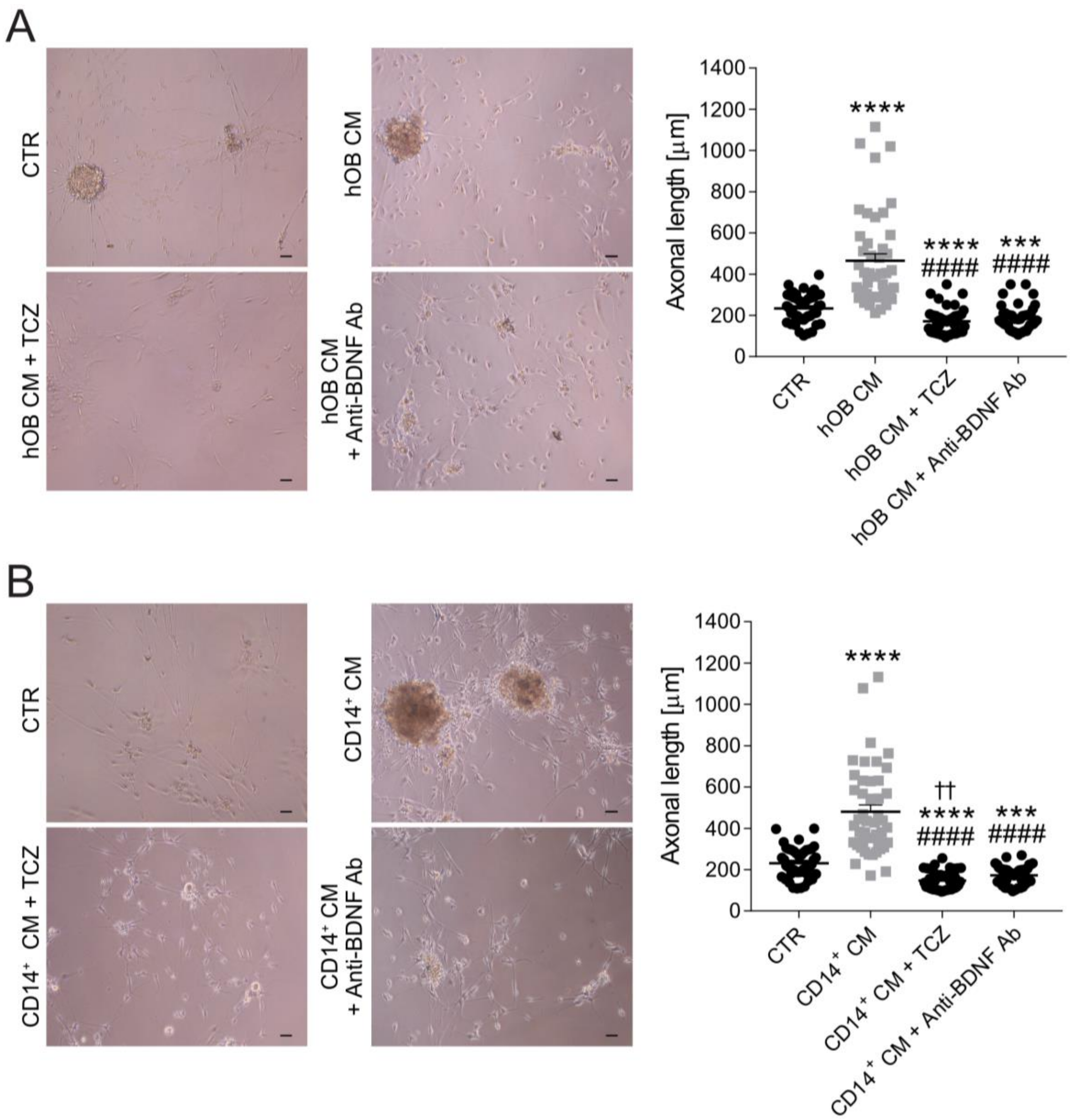

Supplementary Fig. S11. Axonal outgrowth promoted by osteoblasts and monocytes is mediated by IL-6 and BDNF.

(A) DRG neurons exposed for 72 h to osteoblast CM (hOB CM), with or without anti-IL-6 antibody (Tocilizumab, TCZ), or anti-BDNF antibody (Anti-BDNF Ab) (20× lens, scale bar 100 μm). Right, quantification of axon length (mean ± SE, 3 inter-assay and 15 intra-assay replicates, N = 45, $^{****}p < 0.0001$ vs CTR, $^{\#\#\#\#}p < 0.0001$ vs hOB CM). **(B)** DRG neurons exposed for 72 h to $CD14^+$ monocytes-CM ($CD14^+$ CM), with or without TCZ or Anti-BDNF Ab (20× lens, scale bar 100 μm). Right, quantification of axon length (mean ± SE, 3 inter-assay and 15 intra-assay replicates, N = 45, $^{****}p < 0.0001$ vs CTR, $^{\#\#\#\#}p < 0.0001$ vs $CD14^+$ CM, $^{\dagger\dagger}p < 0.001$ vs $CD14^+$ CM + Anti-BDNF Ab).

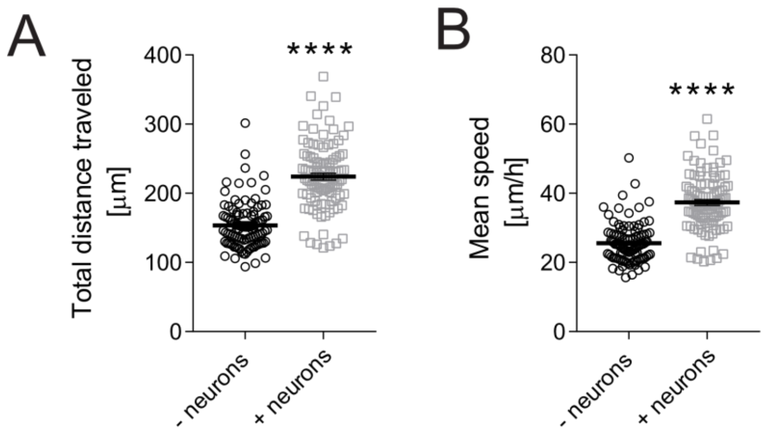

Supplementary Fig. S12. Effect of DRG Neurons on 143B OS Cell Migration.
(A) Quantification of the total distance travelled by 143B OS cells co-cultured with or without DRG neurons for 24 hours (mean ± SE, 2 inter-assay and 3 intra-assay replicates, N = 6, **** $p < 0.0001$ vs. -neurons). (B) Quantification of the mean migratory speed of 143B OS cells co-cultured with DRG neurons compared to monoculture (mean ± SE, 2 inter-assay and 3 intra-assay replicates, N = 6, ****$p < 0.0001$ vs. -neurons).
